# Seamless Gene Correction in the Human Cystic Fibrosis Transmembrane Conductance Regulator Locus by Vector Replacement and Vector Insertion Events

**DOI:** 10.3389/fgeed.2022.843885

**Published:** 2022-04-06

**Authors:** Shingo Suzuki, Keisuke Chosa, Cristina Barillà, Michael Yao, Orsetta Zuffardi, Hirofumi Kai, Tsuyoshi Shuto, Mary Ann Suico, Yuet W. Kan, R. Geoffrey Sargent, Dieter C. Gruenert

**Affiliations:** ^1^ Department of Otolaryngology–Head and Neck Surgery, University of California, San Francisco, San Francisco, CA, United States; ^2^ Department of Medicine, University of California, San Francisco, San Francisco, CA, United States; ^3^ Center for Stem Cell and Regenerative Medicine, Brown Foundation Institute of Molecular Medicine, University of Texas Health Science Center at Houston, Houston, TX, United States; ^4^ Department of Molecular Medicine, Graduate School of Pharmaceutical Sciences, Kumamoto University, Kumamoto, Japan; ^5^ Department of Molecular Medicine, University of Pavia, Pavia, Italy; ^6^ Institutes for Human Genetics, University of California, San Francisco, San Francisco, CA, United States; ^7^ Department of Laboratory Medicine, University of California, San Francisco, San Francisco, CA, United States; ^8^ GeneTether Inc., San Lorenzo, CA, United States; ^9^ Department of Pediatrics, University of Vermont College of Medicine, Burlington, VT, United States

**Keywords:** cystic fibrosis, iPS cells, seamless gene correction, homology directed repair, non-homologous end joining, vector replacement event, vector insertion event, intrachromosomal homologous recombination

## Abstract

**Background:** Gene correction *via* homology directed repair (HDR) in patient-derived induced pluripotent stem (iPS) cells for regenerative medicine are becoming a more realistic approach to develop personalized and mutation-specific therapeutic strategies due to current developments in gene editing and iPSC technology. Cystic fibrosis (CF) is the most common inherited disease in the Caucasian population, caused by mutations in the CF transmembrane conductance regulator (CFTR) gene. Since CF causes significant multi-organ damage and with over 2,000 reported *CFTR* mutations, CF patients could be one prominent population benefiting from gene and cell therapies. When considering gene-editing techniques for clinical applications, seamless gene corrections of the responsible mutations, restoring native “wildtype” DNA sequence without remnants of drug selectable markers or unwanted DNA sequence changes, would be the most desirable approach.

**Result:** The studies reported here describe the seamless correction of the *W1282X CFTR* mutation using CRISPR/Cas9 nickases (Cas9n) in iPS cells derived from a CF patient homozygous for the *W1282X* Class I *CFTR* mutation. In addition to the expected HDR vector replacement product, we discovered another class of HDR products resulting from vector insertion events that created partial duplications of the *CFTR* exon 23 region. These vector insertion events were removed *via* intrachromosomal homologous recombination (IHR) enhanced by double nicking with CRISPR/Cas9n which resulted in the seamless correction of *CFTR* exon 23 in CF-iPS cells.

**Conclusion:** We show here the removal of the drug resistance cassette and generation of seamless gene corrected cell lines by two independent processes: by treatment with the PiggyBac (PB) transposase in vector replacements or by IHR between the tandemly duplicated *CFTR* gene sequences.

## Introduction

Cystic fibrosis (CF) is the most common inherited disease in the Caucasian population, caused by mutations in the CF transmembrane conductance regulator (*CFTR*) gene (Riordan et al., 1989); over 2,000 disease-causing *CFTR* mutations have been reported (CFTR2, 2021). CF patients typically exhibit mucus accumulation, which causes multi-organ damage in many tubular organs such as lungs, pancreas, liver, kidneys, and intestine. Especially the airway and lungs in CF patients have severe pathologies caused by abnormal mucus accumulation and hyper-inflammation accompanied by bacterial infections (O’Sullivan and Freedman, 2009; Hartl et al., 2012). Therefore, one treatment for CF patients would require a comprehensive strategy that both corrects the underlying genetic defect and repairs/regenerates damaged tissues. With this idea, we and others have been developing and have achieved *CFTR* mutation correction in iPSCs derived from a patient homozygous for the *F508del CFTR* mutation, found in around 70% of CF patients. Those corrected CF-iPS cells were ultimately differentiated *in vitro* into airway epithelium and showed the restoration of CFTR function (Crane et al., 2015; Firth et al., 2015; Shingo Suzuki et al., 2016; Ruan et al., 2019; Hawkins et al., 2021). Such cells could be used for autologous transplantations to treat CF in the future.

The *W1282X CFTR* mutation is caused by a G > A transition at codon 1,282 in exon 23 to generate a Tryptophan to stop codon (TGG > TGA) and *W1282X CFTR* cells synthesize a truncated CFTR protein which exhibits little or no function. The *W1282X* mutation is one of the most common *CFTR* mutations following the *F508del* mutation and approximately 2% of patients with CF have at least 1 copy of the *W1282X* mutation (CFTR2, 2021). The mutation is found at a high frequency among Ashkenazi-Jewish and Middle Eastern populations and is observed in approximately 20% of CF patients from those populations (Shoshani et al., 1992; Bobadilla et al., 2002; Castellani et al., 2008).

Gene editing technologies that enable the potential for treating genetic disease have rapidly developed after the engineering of DNA sequence-specific nucleases such as ZFNs (Miller et al., 2007), transcription activator-like effector nucleases (TALENs) (Cermak et al., 2011; Miller et al., 2011), and the RNA-guided CRISPR/Cas9 nuclease (Cong et al., 2013; Mali et al., 2013). These nucleases introduce double-strand breaks (DSBs) at a targeted DNA sequence, which enhances genome modifications by utilizing cellular DNA repair processes such as homology directed repair (HDR) and non-homologous end joining (NHEJ). When considering clinical applications, seamless gene corrections of the responsible mutations would be the most desirable approach. Recently, two approaches have been used to achieve seamless corrections in iPS cells. One approach utilizes HDR for gene targeting, replacing a mutation(s) with corrective donor DNA, followed by treatment with the PiggyBac transposase system to remove drug selectable markers. In this approach, HDR between donor DNA sequences and a genomic target inserts a drug selectable marker that can be seamlessly removed upon the expression of PB transposase (PBase). This two-step system with the replacement and excision has been used to target a variety of genes and cell types (Yusa et al., 2011; Ye et al., 2014). Another approach is a one-step strategy using short single-strand or double-strand DNA oligonucleotides, as homologous donor DNA templates followed by the clonal isolation of modified clones by limiting dilution enrichment methods (Miyaoka et al., 2014; Sargent et al., 2014; Ruan et al., 2019). The latest research using Cas9 ribonucleoprotein (RNP) and recombinant adeno-associated virus (AAV) vector as donor DNA shows highly efficient targeting in iPSCs, which has a great potential to improve upon the one-step approach (Martin et al., 2019).

**FIGURE 1:**
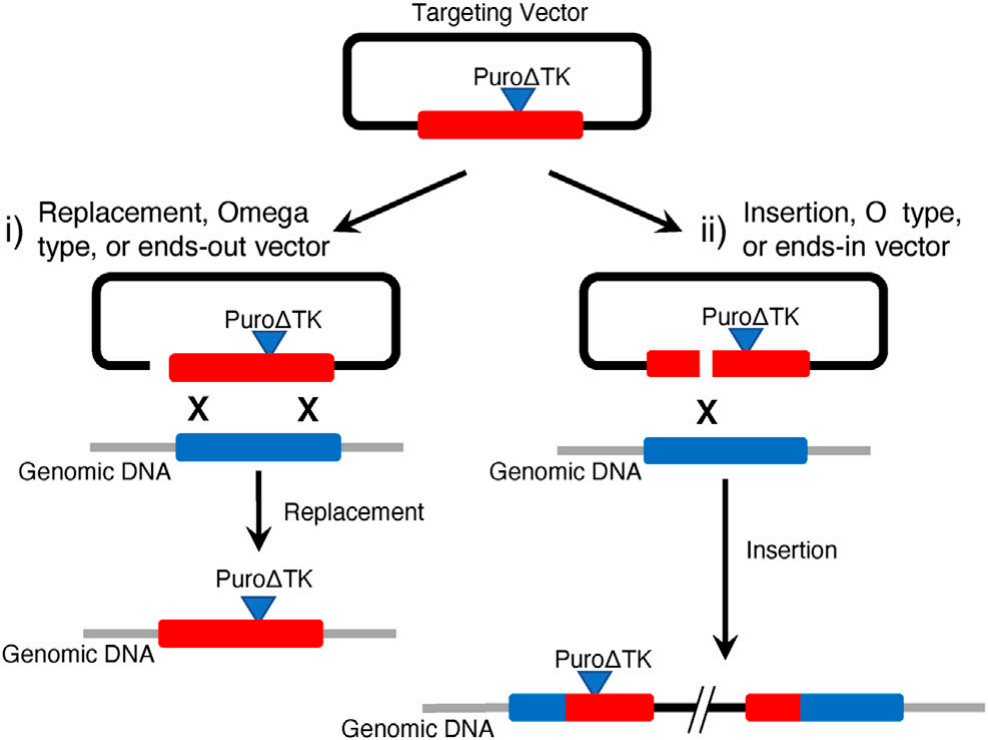
Classical Gene Targeting Strategies. This illustrates expected HDR products observed in mammalian cell lines using i) replacement vectors linearized at the border of homology arms and plasmid backbone and ii) insertion vectors linearized within homology arms. Vector replacement events result in replacement of genomic target DNA with homologous vector DNA sequences, including any drug selectable markers that might be present. Vector insertion events characteristically incorporate the entire plasmid backbone to create a tandem duplication of targeted genomic sequence and any drug selectable markers that might be present. In this example, the *Puro∆TK* drug selectable cassette is present in the vector insertion event 5′ duplication but would be predicted to be found in the 3′ duplication if the targeting vector was linearized 3′ of the vector *Puro∆TK* cassette.

Classical eukaryotic cell gene targeting strategies with mammalian somatic cells and mouse embryonic stem cells, before the development of programmable ZFN, TALEN, and CRISPR/Cas nucleases, could be used to engineer cells containing two different HDR recombination products (Figure 1). Gene replacement events were the prevalent desired HDR product from targeting vectors linearized between vector backbone sequence and DNA sequences homologous to the genomic target. These are sometimes referred to as replacement-, ends-out-, or omega-type-vectors (Figure 1). With replacement vectors, recombination between the vector donor DNA sequence and the cellular target would result in replacement of target genomic sequences with donor DNA. If drug selectable markers are present in the donor DNA they would also be included in the HDR product. In order to create seamless, or almost seamless, gene corrections the drug selectable markers would be removed in a second step, often by using Cre/loxP recombination (Sauer and Henderson, 1989). Alternatively, if targeting vectors are linearized within the homologous donor DNA sequences, referred to as insertion-, ends-in- or O-type-vectors, then HDR products from targeting vector insertion/integration could be recovered inserting the entire targeting vector sequence and generating a partial gene duplication (Figure 1).

In this study, to demonstrate seamless gene correction in human iPSCs derived from a CF patient homozygous for the *W1282X* Class I *CFTR* mutation, we performed a two-step approach: first by using CRISPR/Cas9 nickases (CRISPR/Cas9n) to generate DSBs and catalyze gene correction of the *W1282X* mutation by HDR with a wild type *CFTR* exon 23 targeting vector that contained a puromycin resistance-Herpes virus thymidine kinase (*PuroΔTK*) expression cassette. We chose to use CRISPR/Cas9n in this study to minimize off-target events (Ran et al., 2013a). The seamless removal of the *PuroΔTK* gene cassette from clones containing a gene corrected *W1282X-*wt allele was performed by transfecting *W1282X*-corrected cells with a PBase expression cassette and selecting for loss of the *PuroΔTK* cassette. Further analysis of the original puromycin resistant clonal isolates revealed the surprising presence of cells with vector insertion events containing gene duplications of exon 23. These vector insertion cell lines allowed an alternative strategy to carry out seamless gene correction by CRISPR/Cas9n stimulation of intrachromosomal homologous recombination (IHR) similar to IHR stimulated by the I-SceI endonuclease (Sargent et al., 1997; Donoho et al., 1998; Liang et al., 1998; Sargent et al., 2000). This novel approach would expand the potential to generate seamless HDR targeted cell lines in both basic research and gene targeting therapies. To our knowledge this is one of the first examples of HDR vector insertion events using CRISPR/Cas9 and detection of this class of events in human iPS cells. HDR vector insertion events may represent a largely unrecognized, but useful, class of recombination products present in nuclease-stimulated gene editing experiments.

## Results

### *W1282X CFTR* Gene Targeting in Cystic Fibrosis Patient iPSCs *via* Homology Directed Repair

Patient-specific pluripotent cell lines offer the potential for differentiation into cells and tissues that could be used in autologous transplants. However, correcting mutations that cause genetic disease in iPS cells can be technically challenging and can involve drug selectable markers that need to be removed before differentiation into clinically useful products. The CF-iPS cells used in these studies are homozygous for the *W1282X* mutation (CF2-iPSCs) and were previously generated in the Gruenert laboratory and validated for genotype and iPS cell pluripotent characteristics prior to gene editing experiments (Supplemental Materials: Supplementary Figure S1). The presence of the homologous *W1282X/W1282X* mutation in *CFTR* exon 23 in two representative iPSC clonal isolates, clone 3 and clone 9, was confirmed by DNA sequence analysis (Supplemental Materials: Supplementary Figure S1A). Cytogenetic analysis of clone 3 and clone 9 between P8.9–P8.12 (where passage number PX.Y.etc., = X passages before transduction/reprogramming. Y passages since candidate colony isolation) showed a normal diploid male karyotype (46, XY) (Supplemental Materials: Supplementary Figure S1B). Immunocytochemical analysis showed that clone 3 and clone 9 iPSCs expressed the pluripotent markers NANOG, SSEA4, TRA1–60, and TRA1–81 (Supplemental Materials: Supplementary Figure S1C). Pluripotency was further demonstrated *in vitro* by formation of embryoid bodies (EBs) and immunocytochemical detection of the three germ layer markers α-fetoprotein (AFP, endoderm), *α*-smooth muscle actin (SMA, mesoderm), and *β*-tubulin 3 (TUJ1, ectoderm) (Supplemental Materials: Supplementary Figure S1D). Thus, we used clones 3 and 9, designated as CF2-iPS3 and CF2-iPS9 cell lines in this study.

Homology directed repair (HDR) between gene targeting vectors and their chromosomal targets can result in at least two classes of recombination events, vector replacement events or vector insertion events, depending on the site of vector linearization (Valancius and Smithies, 1991; Hasty et al., 1994). Upon emergence of site-specific nucleases to induce DSBs in genomic DNA, the focus by most laboratories has been on creating vector replacement events through HDR pathways. Recently, targeted vector insertions at chromosomal targets by NHEJ pathways have also been reported, including insertion of whole targeting plasmid DNA, [PITCh (Microhomology-mediated end-joining, MMEJ) (Nakade et al., 2014), ObLiGaRe (NHEJ) (Maresca et al., 2013)], homology-independent targeted integration (HITI) (Keiichiro Suzuki et al., 2016) and insertion in ESCs [NHEJ (He et al., 2016)]. The studies described here demonstrate that gene targeting using non-linearized vectors can result in not only vector replacement, but also vector insertion events by HDR when DSBs are introduced at targeted sites.

### Homology Directed Repair Vector Replacement Events

**FIGURE 2:**
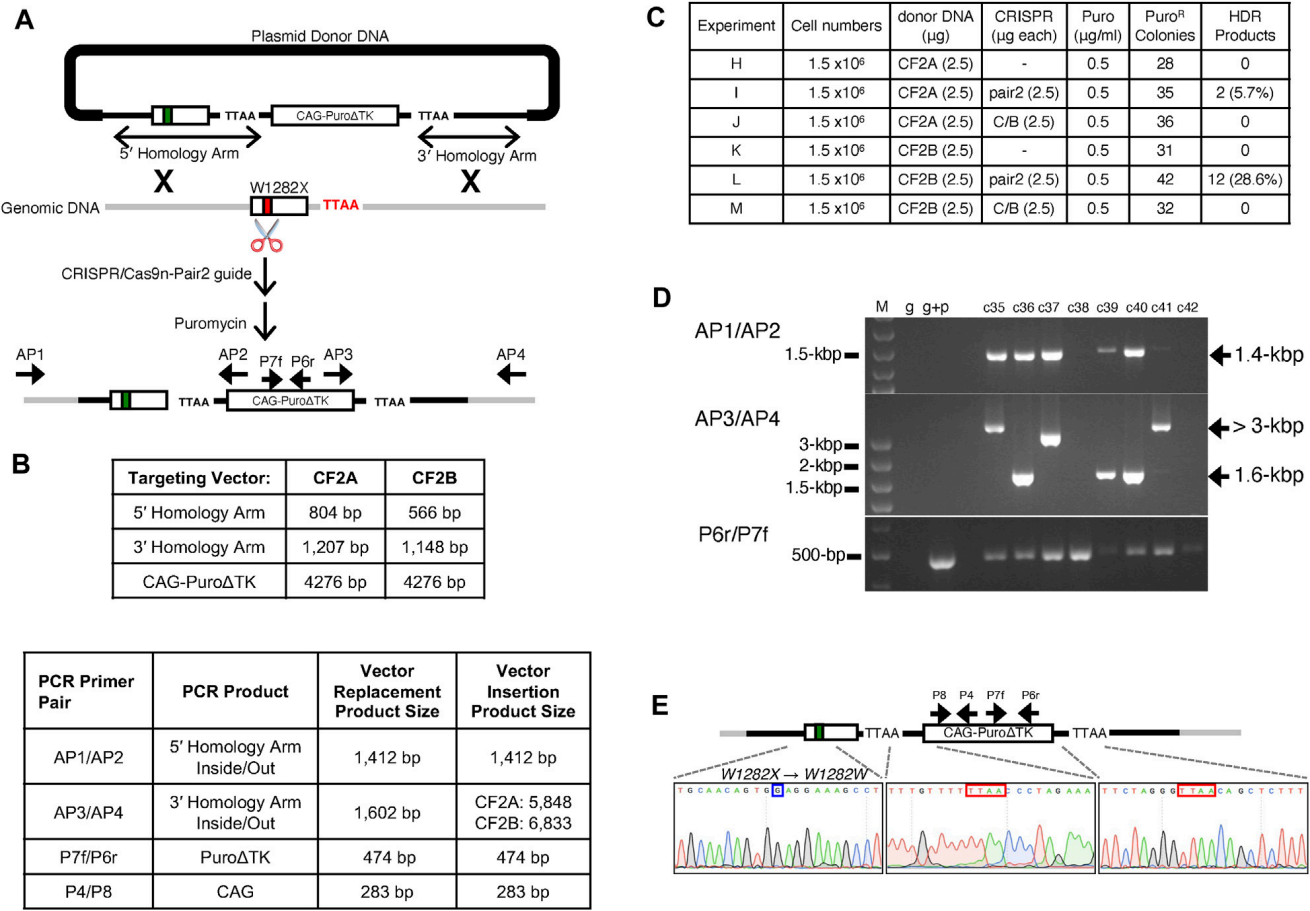
Characterization of Vector Replacement Events in CF2-iPS3 Cells **(A)** Illustration of targeting strategy *via* HDR with donor DNA carrying *Puro∆TK* cassette, described in methods. PCR primers (AP1/AP2, AP3/AP4, and P6r/P7f) used to confirm successful HDR are illustrated next to the annealing sites. AP1/AP2; intron at upstream of *CFTR* exon 23 through the 5′ end of the *Puro∆TK* cassette. AP3/AP4; 3′ end of *Puro∆TK* cassette through intron at downstream of exon 23. P6r/P7f; Puromycin resistant gene at Puro∆TK cassette. In this figure and subsequent figures the medium black line represents vector DNA sequences homologous to the exon 23 region, grey lines represent genomic DNA and the heavy black line represents plasmid backbone DNA sequence. Exon 23 is represented by an open rectangle with the *W1282X* allele represented by a red box and the *W1282X*-wt corrected allele is represented by a green box in the exon 23 rectangle. **(B)** Homology arm, Puro∆TK, and diagnostic PCR amplicon lengths. **(C)** Experimental transfection details for drug selections and HDR efficiencies. **(D)** HDR products as assayed by PCR are also shown in Supplemental materials Supplementary Figure S3, with agarose gel pictures for represented colonies. M; 1 kb or 100 bp DNA ladder plus, g; CF2-iPS3 genomic DNA, g + p; the mixture of CF2-iPS3 genomic DNA and donor DNA. High molecular weight (HMW) fragments (>3 kbp) observed in AP3/AP4 PCR were further investigated in Figure 4, and Supplementary Figures S5, S6. **(E)** Sequence chromatogram showing the 5 and 3′ junctions of the Puro∆TK cassette (shown in red box) and *W1282X* mutation site in *CFTR* exon 23 (shown in blue box) in the Ic8 clone. Sanger sequencing was performed on AP1/AP2 and AP3/AP4 amplicons to sequence only targeted allele sequences.

Two gene-targeting vectors were constructed with different lengths of homology to the genomic target. Both vectors contain a *PuroΔTK* drug selection cassette flanked by DNA sequences homologous to the *CFTR* exon 23 region and both vectors contain the TTAA repeat found in intron 23 for excision of the *PuroΔTK* cassette by expression of the PiggyBac transposase (PBase). The CF2A targeting vector contains a total of 1,370-bp of *CFTR* homologous sequence with a 5′ arm of 804-bp homologous *CFTR* sequence and a 3′ arm of 566-bp homology to the *CFTR* exon 23 region. The CF2B targeting vector contains 2,355-bp of *CFTR* homologous sequence with a 5′ arm of 1,207-bp and 3′ arm of 1,148-bp of homology, respectively (Figures 2A,B).

For the induction of CRISPR/Cas9n double strand breaks in the *W1282X*-mutant *CFTR* DNA sequence, but not *W1282X*-wild type (wt) *CFTR* sequence**,** four pairs of guide RNA sequences (gRNAs) were designed to cleave at specific genomic DNA sequences adjacent to, or overlapping, the *W1282X*-mutant DNA sequence and were cloned into CRISPR/Cas9n expression vectors. Binding and DNA strand nicking of both members of a CRISPR/Cas9n pair would be required to generate DSBs. Reduced off-target DSB induction has been demonstrated using CRISPR/Cas9n pairs which has the additional advantage of minimizing DSB induction in *W1282X-*wt *CFTR* vector DNA sequences (Ran et al., 2013a).

All four pairs of *CFTR* exon 23 Cas9n gRNAs share the same 5′ gRNA (Supplementary Figure S2A) and differ only in the 3′ gRNA, complementary to the opposite DNA strand. In order to introduce DSB only in DNA with *W1282X*-mutant sequence, we designed the 3′ gRNA for pair-2 and pair-3 to overlap with the *W1282X* G > A transition mutation. The pair-2w 3′ gRNA is complementary to the *W1282X*-wt *CFTR* DNA sequence and the pair-1 3′ gRNA is complementary to DNA sequence downstream of the *W1282X*-mutant *CFTR* DNA sequence and thus can bind and cut *W1282X-*wt or *W1282X*-mutant sequences (Supplemental Materials: Supplementary Figure S2A).

The nickase gRNA pairs were assayed for nuclease efficiency and mutation specificity using the T7 endonuclease I (T7E1) assay with genomic DNA isolated CRISPR/Cas9n/gRNA transfections of CFBE41o- cells (*W1282X-*wt *CFTR* sequence) (Vouillot et al., 2015). The pair-2 and pair-3 gRNA (*W1282X*-mutation-specific) induced approximately 1.3 and 9.9% NHEJ induction, respectively. Because the *CFTR* exon 23 sequence in CFBE41o- cells is wt for the *W1282X* mutations, pair 2 and pair 3 gRNA were expected to have little, or no, on-target cutting. In order to confirm the specificity of the pair-2 gRNA molecules and their ability to discriminate against *W1282X-*wt sequence, we generated pair-2w gRNA that is complementary to the *W1282X*-wt sequence. Transfection of CFBE41o- cells with pair-2w gRNA resulted in approximately 11.1% NHEJ induction. Since this result suggests that pair-2 gRNA targeting could achieve allele specific discrimination between *W1282X*-mutant and *W1282X*-wt sequences (Supplemental Materials: Supplementary Figure S2B), thus minimizing DSB induction in target vector DNA sequences, pair-2 gRNA was used for targeting experiments in CF2-iPSCs.

A similar approach as above was used to validate allele-specific gRNA to cleave genomic DNA, but not DNA containing the *PuroΔTK* cassette. Two CRISPR/Cas9n pairs (pair-A/B and pair-C/B) were designed to target adjacent to a TTAA sequence where the *PuroΔTK* cassette is present in donor DNA and would be inserted (Supplemental Materials: Supplementary Figure S2A) into the *CFTR* region on gene targeting by vector replacement. In this approach the A and C gRNA sequences would not have complementarity to the *PuroΔTK*. However, neither the A/B nor the C/B gRNA pairs demonstrated nuclease activity on target *CFTR* sequences after transfection into CFBE41o- cells and analysis by T7E1 assay (Data not shown).

For gene targeting experiments to correct the *W1282X*-mutation in *CFTR* exon 23, CRISPR/Cas9n-pair-2 gRNA expression vectors were co-transfected with either unlinearized targeting vector CF2A or unlinearized targeting vector CF2B into CF2-iPS3 cells (Figure 2A). Transfected cells were selected with Puromycin for 2 weeks, and then well-isolated puromycin resistant (puromycin^R^) colonies were picked and assayed for vector replacement events by an inside-out PCR strategy with one PCR primer complimentary to the *Puro∆TK* cassette (AP2 for 5′ end or AP3 for 3′ end of the *Puro∆TK* cassette) and the other PCR primer complementary to genomic DNA sequence outside of the homology arms in donor DNA (AP1 for the upstream of 5′- or AP4 for 3′-arm). The AP1/2 and AP3/4 primer pairs amplify a 1,412-bp or a 1,602-bp PCR fragment, respectively, from HDR replacement-targeted alleles (Figures 2A,B, Supplemental Materials: Supplementary Figure S3).

There were 28 (CF2A) and 31 (CF2B) puromycin^R^ colonies isolated from transfection of donor DNA alone (Figure 2C Expt H and K). These are presumably randomly integrated vectors due to the lack of AP1/2 or AP3/4 PCR products in cells positive by PCR for the PuroΔTK cassette (Supplemental Materials: Supplementary Figure S3; Expts H and K). However, co-transfection of the CRISPR/Cas9n-pair-2 with the CF2A targeting vector containing shorter homology arms (Figure 2C Expt I) or with the CF2B targeting vector containing longer homology arms (Figures 2C,D Expt L) resulted in HDR targeting efficiencies of 5.7% (2 AP1/2 and AP3/4 PCR^+^ colonies/35 *PuroΔTK* colonies, Figure 2C Expt I) or 28.6% (12 AP1/2 and AP3/4 PCR^+^ colonies /42 *PuroΔTK* colonies, Figure 2C Expt L), respectively (Supplemental Materials: Supplementary Figure S3) as determined by PCR products for both AP1/2 and AP3/4 primers. On the other hand, co-transfection of the CRISPR/Cas9n-pair-C/B did not show any targeting at the *CFTR* exon 23 locus (Figure 2C and Supplemental Materials: Supplementary Figure S3; Expts J and M), consistent with the negative results of the T7E1 assay for pair-C/B gRNA.

One candidate clone from Expt I, clone 8 (Ic8), was used for further characterization of the HDR product and used for generation of seamless *W1282X*-wt corrected cells by excision of the *PuroΔTK* cassette by PBase expression. To demonstrate that clone Ic8 resulted from HDR vector replacement between genomic DNA and donor DNA, and was not due to NHEJ integration of vector DNA sequences, the junctions between vector DNA and the genomic target for the targeted *W1282X* alleles were genotyped by sequencing the AP1/2 and AP3/4 PCR products. Gene correction of one *W1282X-*mutant *CFTR* sequence and the insertion of the *Puro∆TK* cassette flanked by a TTAA repeat sequence were likewise confirmed by DNA sequencing (Figure 2E) of AP1/2 and AP3/4 PCR products.

### Excision of the *PuroΔTK* Cassette by Expression of PBase

**FIGURE 3:**
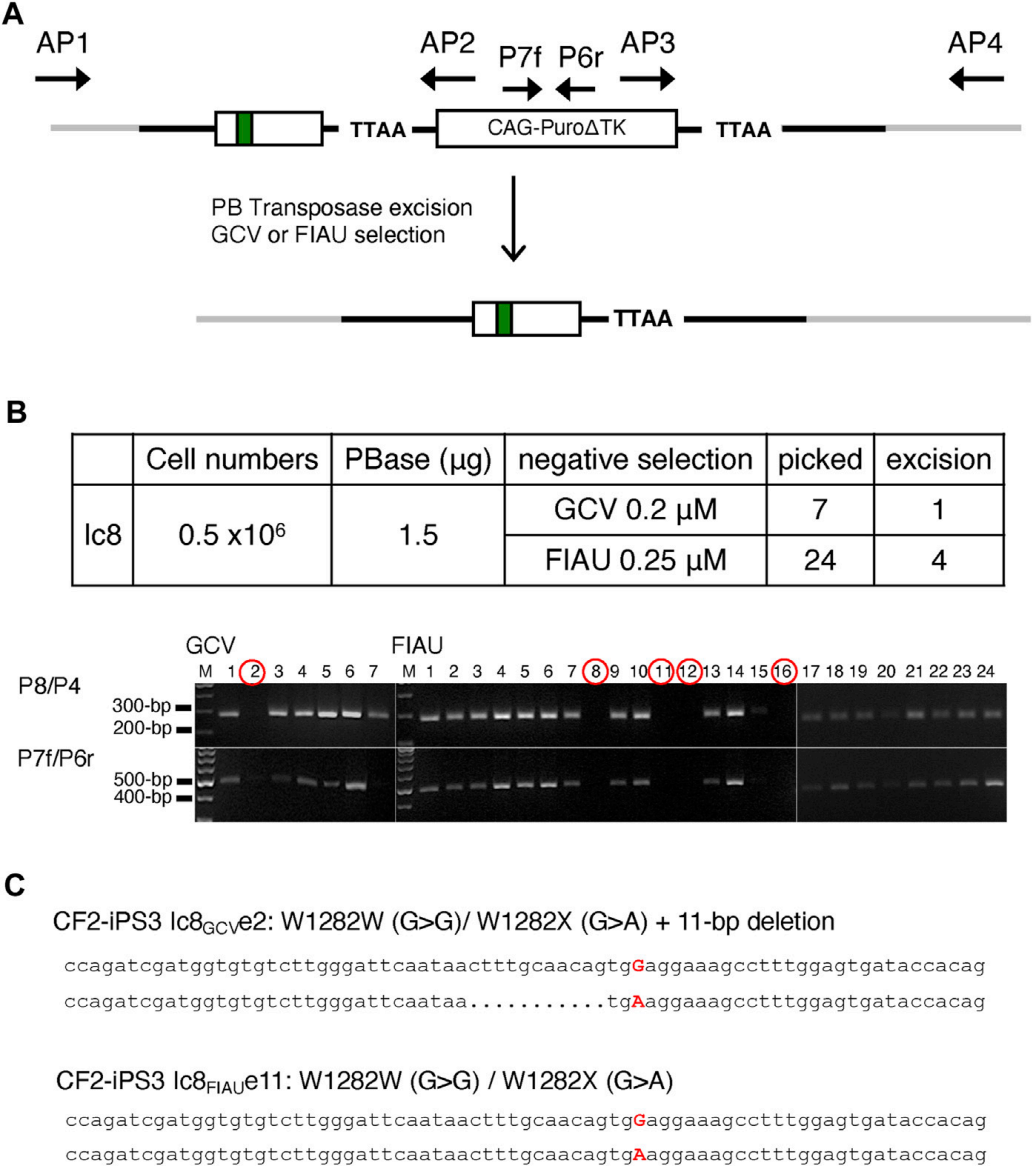
PBase Removal of the *Puro∆TK* Cassette from Vector Replacement Cell Lines. **(A)** Graphic illustration of the *Puro∆TK* cassette excision. **(B)** Colonies surviving GCV or FIAU selection excision were confirmed *as Puro∆TK* cassette negative clones by PCR using P8/P4 and P7f/P6r primers to generate the PCR results as shown in gel pictures. M; 100 bp DNA ladder plus. **(C)** Sequence of excised clones screened from PBase-transfected Ic8 clones selected with 0.2 μM of GCV (Ic8GCVe2, top clone) or with FIAU (Ic8FIAUe11, bottom clone). Adenine (A) and Guanine (G) capitalized and highlighted in red representing *W1282X* mutation (G > A) and the correction (A > G), respectively. Dot represents deleted nucleotide.

Excision of the *PuroΔTK* drug selection cassette in Ic8 cells was achieved by transient expression of the PB transposase, followed by negative selection with Ganciclovir (GCV) or Fialuridine (FIAU) to select for cells where the *PuroΔTK* cassette was excised (Figure 3A). Seven independent GCV-resistant (GCV^R^) colonies and 24 FIAU resistant (FIAU^R^) colonies were isolated, and removal of the *PuroΔTK* cassette was confirmed by PCR with P8/P4 and P7f/P6r PCR primer pairs (Figure 3B). One of the seven GCV^R^ clones (clone 2) was negative by PCR for the *PuroΔTK* cassette and four of the 24 FIAU^R^ clones (clones 8, 11, 12, and 16) were negative by PCR for the *PuroΔTK* cassette (Figure 3B). Seamless excision of the *PuroΔTK* cassette was confirmed by DNA sequencing of AP1/AP4 PCR products from genomic DNA of GCV^R^ clone 2 (referred to as Ic8_GCV_e2) and the FIAU^R^ clone 11 (referred to as Ic8_FIAU_e11) (Figure 3C). The Ic8_FIAU_e11 clone genomic DNA shows a *W1282X*-wt corrected allele and an uncorrected *W1282X-*mutant allele, with precise excision of the *PuroΔTK* cassette. The Ic8_GCV_e2 genomic DNA sequence shows a *W1282X*-wt corrected allele and an uncorrected *W1282X-*mutant allele with a 11-bp deletion that overlaps the CRISPR/Cas9n-pair2 nicking sites. Since the AP1/AP4 PCR primers would amplify the maternal and paternal *CFTR* exon 23 sequences, the DNA sequencing results confirm that only one allele has been corrected in the Ic8 cell line.

The gene corrected CF2-iPS3 Ic8_FIAU_e11 and Ic8_GCV_e2 clones maintained their pluripotency and normal karyotype. Clones Ic8_GCV_e2 and Ic8_FIAU_e11 expressed the pluripotent markers NANOG, SSEA4, TRA1-60, and TRA1-81 and upon differentiation expressed the three germ layer markers AFP, SMA, and TUJ1 (Supplemental Materials: Supplementary Figure S4A). In addition, both modified CF2-iPS3 cells clones had a normal male karyotype (Supplemental Materials: Supplementary Figure S4B).

### Homology Directed Repair Vector Insertion Events

**FIGURE 4:**
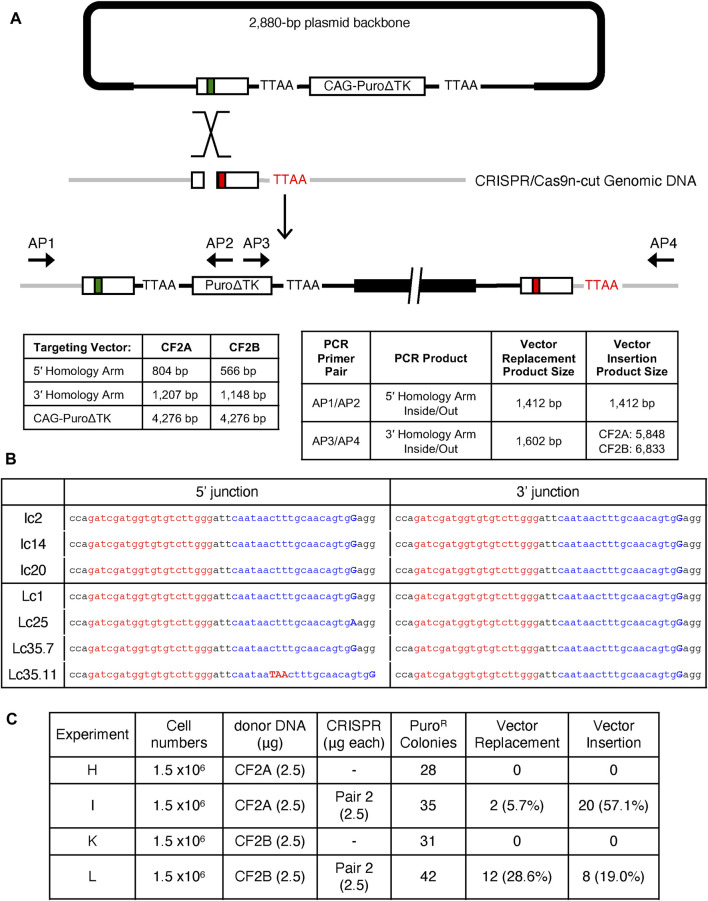
Identification of Vector Insertion Events. **(A)** Schematic illustration of site-specific insertion event with donor DNA backbone. AP3/AP4 PCR amplifies 5,848-bp product from CF2A donor transfection and 6,833-bp product from CF2B donor transfection, which are confirmed in Figure 2D, and Supplemental materials: Supplementary Figure S3. **(B)** Representative sequence alignments at 5′ and 3′ junctions as the consequence of each donor DNA insertion, CF2A (I) or CF2B (L) donor. Lc35 clone initially isolated was a mixed population of Lc35.7 and Lc35.11 cells. Targeting sequences of pair 2 gRNA are shown in red and blue. Adenine (A) and Guanine (G) capitalized and representing W1282X mutation (G > A) and the correction (A > G), respectively. **(C)** Comparison of frequencies between site-specific vector replacement and insertion events. The frequency of each event was calculated with the number of positive clones out of the number of picked and analyzed Puro^R^ clones.

Vector insertion gene targeting products have been observed in classical gene targeting experiments with immortalized cell lines and mouse embryonic stem cell lines using ends-in targeting vectors (Figure 1; Valancius and Smithies, 1991; Hasty et al., 1994). Based on previous gene targeting experiments in our laboratory, suggesting that HDR vector insertion events were an overlooked class of homologous recombinant products in TALEN and CRISPR/Cas9 stimulated gene targeting (K Chosa, S Suzuki, RG Sargent, unpublished data), we reanalyzed the puromycin^R^ clones from experiments I and L that were excluded as candidate vector replacement events as identified by the initial AP1/AP2 inside-out PCR strategy. While the AP1/AP2 PCR product is diagnostic for all potential HDR events, it alone would not distinguish between vector replacement and vector insertion gene targeting products. Since vector insertion events would result in duplication of the *CFTR* exon 23 and insertion of the vector backbone, the AP3/AP4 PCR products would be larger than for vector replacement events and predicted to be 5,848-bp for the CF2A targeting vector and 6,833-bp for the CF2B targeting vector (Figure 4A). This is in contrast to vector replacement events that would yield an AP3/AP4 PCR product of 1,602-bp (Figures 2A,B). A high molecular weight AP3/AP4 PCR product was observed for the reassayed puromycin^R^ clones (Figure 2D c35, c37, c41, Supplemental Materials: Supplementary Figure S3B Expt I and Expt L) consistent with the predicted PCR products diagnostic for vector insertion events.

To verify the molecular structure of the putative vector insertion events, we used a nested PCR strategy with gel-purified AP3/AP4 PCR products as template (Supplemental Materials: Supplementary Figure S5A). The predicted sizes of AP3/AP4 PCR products were confirmed, and nested PCR products using primers complementary to the plasmid backbone and *CFTR* DNA sequences confirmed the presence of the vector backbone and homology arms (Supplemental Materials: Supplementary Figures S5B, S5C). Restriction enzyme digestions of the AP3/CF44 PCR products also confirmed the molecular structure of insertion events (Supplemental Materials: Supplementary Figure S6).

DNA sequencing across the CRISPR/Cas9n-pair-2 target sequences at the 5′ and 3′ junctions of genomic and plasmid DNA further demonstrated that insertion events were due to HDR. DNA sequencing across the CRISPR/Cas9n-pair-2 target sequence for both copies of the tandemly duplicated *CFTR* exon 23 for 7 clones showed that only one copy in clone Lc35.11 was not a perfect homologous recombinant product and contained a duplication of TAA DNA sequence (Figure 4B). In 6/7 clones, both of the tandemly duplicated exon 23 alleles contained the *W1282X*-wt corrected DNA sequence whereas clone Lc25 retained the *W1282X*-mutant allele in the upstream duplicated exon 23 gene sequence and a *W1282X-*wt allele in the downstream exon 23 gene duplication.

The reanalysis of puromycin^R^ clones from experiments I and L that were excluded as vector replacement events revealed that the majority of puromycin^R^ clones actually resulted from vector insertion events with the CF2A targeting vector (Figure 4C; 57.1% insertion vs. 5.7% replacement) whereas vector replacement events were more frequent for the CF2B targeting vector (Figure 4C; 28.6% replacement vs. 19.0% insertion). The HDR gene targeting frequency, adjusted to include vector insertion events, relative to puromycin^R^ colonies screened for the CF2A targeting vector was 62.8% HDR events compared to 47.6% for the CF2B targeting vector. The remaining puromycin^R^ colonies not identified as vector replacement or vector insertion events are presumably randomly integrated targeting vectors or vectors integrated in exon 23 that have undergone DNA rearrangements.

### Excision of Vector Insertions From Clone Ic14

**FIGURE 5:**
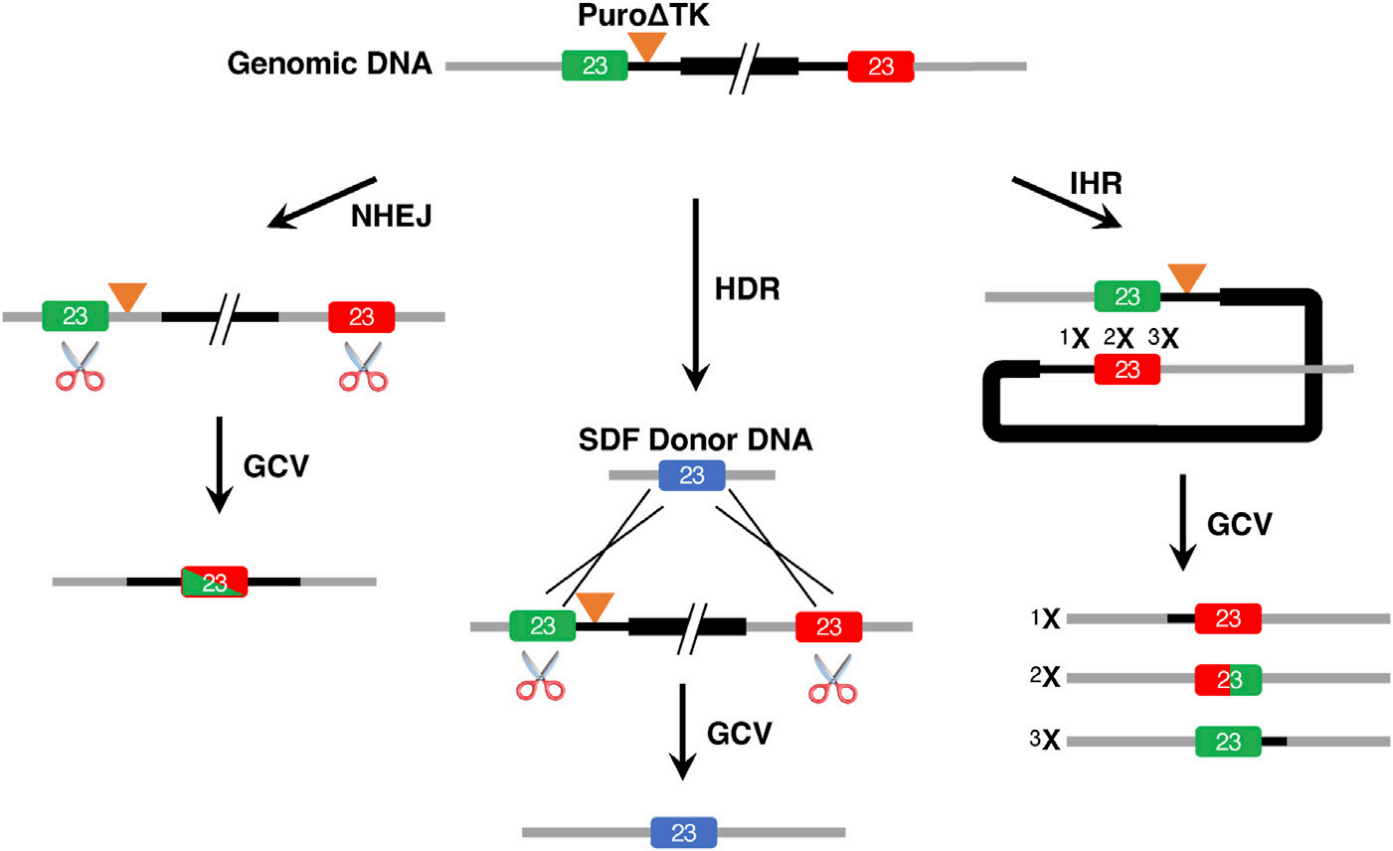
Removal of Vector Insertions by NHEJ, HDR, and IHR. This illustrates the approaches used to generate single-copy exon 23 alleles from vector insertion event cell lines. For this figure the exon colors are used to indicate potential recombination products and do not necessarily represent different *W1282X* alleles.

The tandem duplication of exon 23 found in the vector insertion events allows for several possible strategies for removal of the plasmid backbone and drug selectable markers (Figure 5). Excision of duplicated sequences might be expected to proceed through NHEJ pathways when two DSB are induced within identical duplicated target sequences present in vector insertions (Figure 5 NHEJ and HDR pathways). In this approach, removal of duplicated sequences would result in loss of the *Puro∆TK* cassette and be accompanied by appearance of insertion/deletion mutations, or other gene rearrangements, at repaired DSB junctions. However, if a donor DNA template was available during DSB induction then repair by HDR pathways would be possible (Figure 5). HDR repair by intrachromosomal homologous recombination (IHR) between the duplicated DNA sequences is also a well characterized process when a solitary DSB is induced in or in between duplicated DNA sequences (Figure 5) (Sargent et al., 1997; Donoho et al., 1998; Liang et al., 1998; Sargent et al., 2000). Intrachromosomal homologous recombination between tandemly duplicated sequences would result in loss of the *Puro∆TK* cassette and reconstitute intact genomic target DNA sequence at the DSB induction target sequence to create a seamless gene correction (Figure 5). The clone Ic14 with a tandemly duplicated exon 23 vector insertion event was used for experiments to test for removal of DNA sequences by these three processes.

**FIGURE 6:**
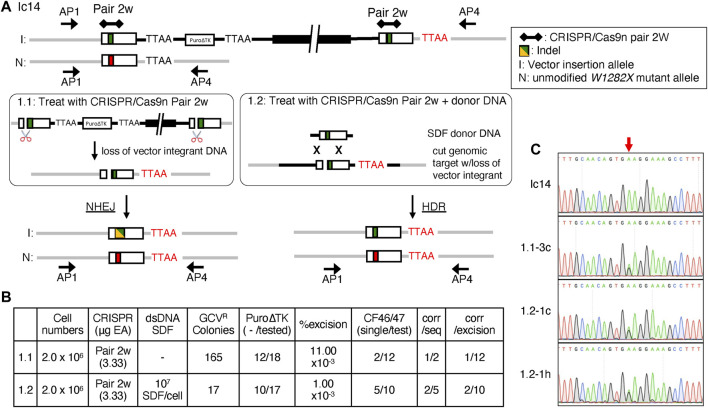
Seamless excision of Vector Insertions by NHEJ and HDR with CRISPR/Cas9n-pair-2w. **(A)** Strategy of excising inserted backbone in order to obtain seamless corrected CF2-iPS3 cells using CRISPR/Cas9n-pair-2w. Both methods were performed using the CRISPR/Cas9n-pair-2w, but in the presence (treatment 1.2) or absence (treatment 1.1) of a homologous donor SDF. The gRNA targeting site is represented by black diamonds and connected by the line for a pair. Indel is indicated by the yellow/green box, the *W1282X*-wt allele is indicated by a green box and the *W1282X*-mutant allele by a red box. I; Vector insertion allele, N; unmodified allele. The red TTAA sequence is the predicted native genome TTAA whereas the black TTAA would be from the targeting vector. The Ic14cell line used had a gene conversion event in the upstream allele to generate a *W128X*-wt allele thus both vector insertion alleles were *W1282X*-wt. **(B)** Comparison of the seamless excision efficiencies in Ic14 clone between treatment 1.2 and 1.1. **(C)** Sequence histogram of AP1/4 amplicon shows single peak of A in *W1282X* site in Ic14 (Pre-excision), and mix peak of A and G in Ic14 1.1-3c, 1.2-1c and 1.2-1h (Post-excision). Red arrow points to *W1282X* mutation site.

Excision of duplicated sequences in Ic14 cells by NHEJ (Figure 6, treatment 1.1) or by HDR with a small DNA fragment (SDF, 1,769-bp *W1282X*-wt *CFTR* DNA fragment) donor DNA template (Figure 6, treatment 1.2) was tested by transfection with the CRISPR/Cas9n-pair-2w which could introduce DSB in either or both copies of the duplicated exon 23 sequences in Ic14 and potentially in the SDF donor DNA sequence. The subsequent loss of intervening plasmid sequences, the *PuroΔTK* cassette, and duplicated *CFTR* genomic sequences by NHEJ, or by HDR with intact donor SDF, would result in GCV-resistant (GCV^R^) colonies (Figure 6A). Nucleofection of 2 × 10^6^ cells with CRISPR/Cas9n-pair-2w expression vector alone resulted in 165 GCV^R^ colonies and only 17 GCV^R^ colonies when co-transfected with the SDF donor DNA template (Figure 6B). Genomic DNA from GCV^R^ clones for each treatment (1.1 and 1.2) were analyzed for excision of the *Puro∆TK* cassette using PCR with primers complementary to *Puro∆TK* sequences (P8/P4 and P7f/P6r primers, Figures 6A,B). In the presumptive recombinant excision clones, the DNA sequence surrounding exon 23 was analyzed by PCR using the CF46/CF47 PCR primer pair to identify clones that potentially arose by NHEJ. Indels created by NHEJ near the CRISPR/Cas9n-pair-2w cut site would result in CF46/CF47 PCR products with amplicon sizes different from the unmodified allele and HDR products. Single CF46/CF47 PCR amplicons diagnostic for seamlessly stitched clones were observed from presumptive recombinant excision clones isolated from CRISPR/Cas9n-pair-2w transfections (Treatment 1.1; Figure 6B; Supplemental Materials: Supplementary Figure S7, clones 3c and 3 g) and were also observed for CRISPR/Cas9n-pair-2w with SDF co-transfection (Treatment 1.2; Figure 6B; Supplemental Materials: Supplementary Figure S7, clones 1.2-1c, 1.2-1e, 1.2-1g, 1.2-1h, and 1.2-2b). Presumptive seamlessly stitched clones were sequenced to confirm the genotype. The AP1/AP4 amplicons were used for sequence analysis because under the PCR conditions used, the AP1/AP4 primer pair amplifies the non-targeted *W1282X-*mutant allele and the recombinant alleles, but not the tandemly duplicated and vector-inserted allele (Figure 6A schematic allele I and Figure 6C, Ic14). Only one clone from treatment 1.1 (Figure 6C, clone 3c) and two clones from condition 1.2 (Figure 6C, clones 1c and 1h) had a DNA sequence demonstrating a seamlessly corrected *CFTR* exon 23 sequence.

**FIGURE 7:**
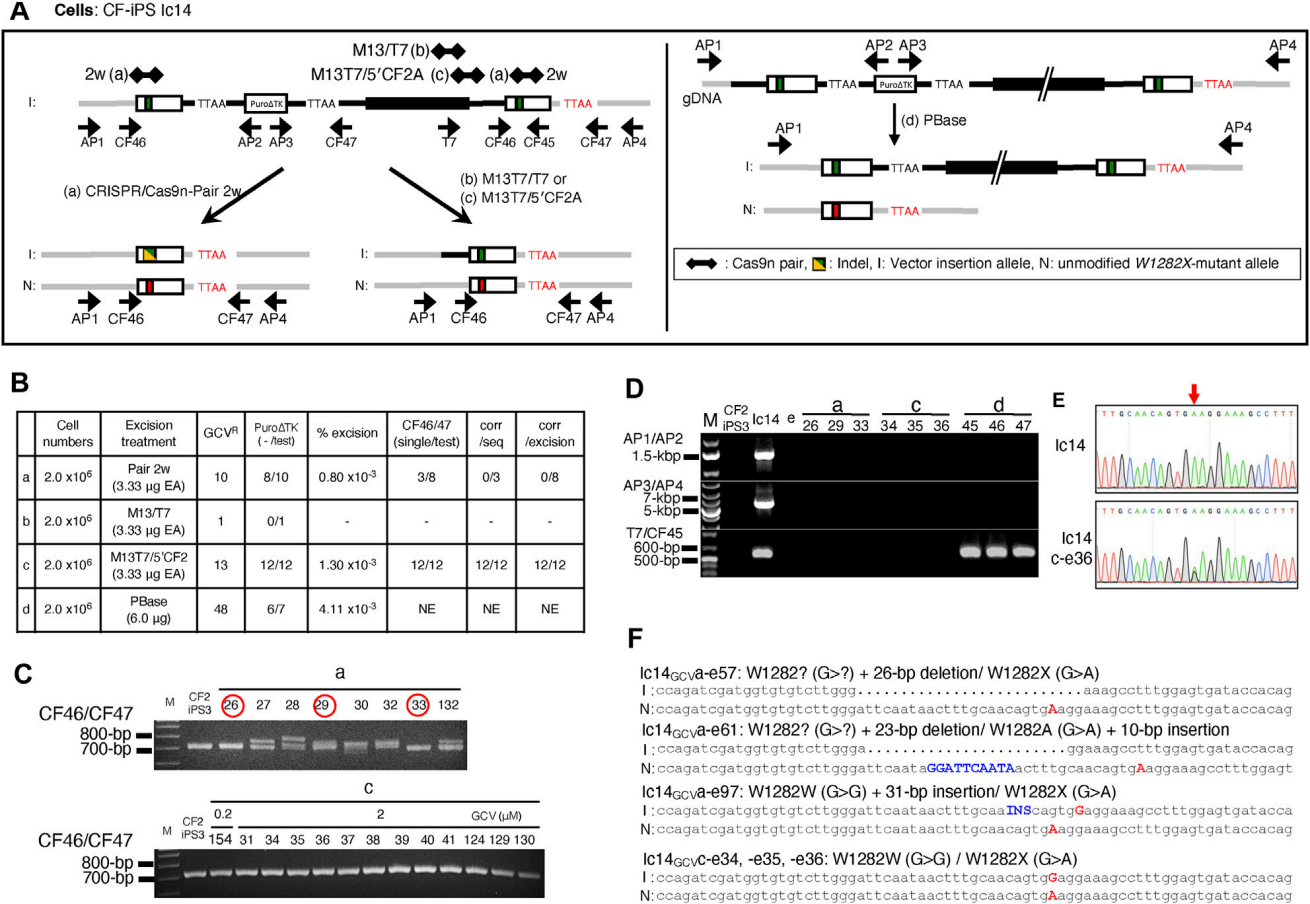
Seamless excision of Vector Insertion DNA backbone with CRISPR/Cas9n stimulated IHR. **(A)** Strategy of excising the inserted plasmid DNA backbone using the CRISPR/Cas9n-gRNAs targeting the junction of plasmid donor backbone and genomic DNA. Newly generated gRNA targeting two regions were used, one of which cut the backbone of plasmid DNA (CRISPR/Cas9n-pair-M13/T7 gRNA, treatment b) and another targeted the junction between backbone and 5′-homology arm of CFTR on donor DNA (CRISPR/Cas9n-pair-M13T7/5′CF2 gRNA, treatment c). For comparison, CRISPR/Cas9n-pair-2w gRNA alone (treatment a) and PBase alone (treatment d) were also tested. **(B)** Summary of the excision efficiencies for each strategy. Ic14 cells (P8.13.14) were transfected four treatments and cells with loss of the *Puro∆TK* selected with GCV. **(C)** Selection of possible seamless excised clones with CF46/47 PCR. Clones a-e26, a-e29, a-e33 (circled in red), and all 12 clones from treatment c had single PCR amplicons indicating precise excision. **(D)** Confirmation of the removal of inserted plasmid DNA backbone DNA sequences using the vector insertion diagnostic primer pairs in representative clones in treatment a, c, and d. **(E)** Sequence histogram of AP1/4 amplicon from Ic14 and Ic14 c-e36. Red arrow points to W1282X mutation site. **(F)** Sequence of representative clones screened from Ic14 clones with excised inserted backbone by treatment a, and c. Red capital letter shows W1282X mutation site. All three Ic14GCVa clones have indel in corrected allele at upstream of W1282X mutation site shown as dot (deletion) or blue letter (insertion). All three Ic14GCVc clones have corrected and uncorrected allele without indels.

Stimulation of IHR between tandemly duplicated *CFTR* sequences, was tested using two different pairs of CRISPR/Cas9n-gRNAs, targeting near (pair-M13/T7) or at (pair-M13T7/5′CF2A) the 3′ junction of plasmid DNA backbone and genomic DNA (Figure 7A). Unlike treatment with CRISPR/Cas9n-pair-2w which could generate two DSB in the duplicated sequences, the CRISPR/Cas9n-pair-M13/T7 and the CRISPR/Cas9n-pair-M13T7/5′CF2A would introduce only one DSB in or in between the duplicated sequences. The efficiency of DSB induction as determined by the T7E1 assay on genomic DNA from Ic14 cell transfections with Cas9n-gRNA expression vectors shows DSB induction with the CRISPR/Cas9n-pair-M13T7/5′CF2A but undetectable DSB induction with the CRISPR/Cas9n-pair-M13/T7. (Supplemental Materials: Supplementary Figure S8). In these experiments, there was no detectable DSB induction in the DNA of Ic14 cells in transfections with the piggyBac transposase vector.

Intrachromosomal homologous recombination between duplicated sequences was tested by transfection of Ic14 cells with Cas9n-gRNA pairs followed by GCV selection for loss of the *Puro∆TK* cassette. Transfection with the Cas9n-pair-M13T7/5′CF2A resulted in 13 GCV^R^ colonies as compared with only 1 colony for Cas9n-pair-M13/T7 transfection (Figure 7B). Transfection of Ic14 cells with Cas9n-pair-2w, which could introduce 2 separate DSB, one in each exon 23 duplication, resulted in 10 GCV^R^ colonies whereas the negative control PBase transfection resulted in 48 GCV^R^ colonies. GCV^R^ colonies from Cas9n-pair-2w, Cas9n-pair-M13/T7, and Cas9n-pair-M13T7/5′CF2, and PBase transfections were examined for loss of the *Puro∆TK* cassette by PCR diagnostic for the *Puro∆TK* sequences with P8/P4 and P6r/P7f PCR primer pairs which confirmed the successful excision in 12/12 Cas9n-pair-M13T7/5′CF2 GCV^R^ colonies and 8/10 Crispr/Cas9n-pair-2w GCV^R^ colonies. The single Cas9n-pair-M13/T7 GCV^R^ colony retained *Puro∆TK* cassette sequences whereas 6/7 GCV^R^ colonies from PBase transfection demonstrated loss of *Puro∆TK* sequences (Figure 7B). Importantly, when we carried out CF46/47 PCR in all *Puro∆TK* cassette-excised clones from CRISPR/Cas9n-pair-M13T7/5′CF2, all clones showed only a single PCR band, while some clones from CRISPR/Cas9n-pair-2w transfections showed multiplex bands (Figure 7C) indicating potential indels from NHEJ. Furthermore, representative *Puro∆TK* cassette-excised clones as well as candidates of seamless corrected clones were tested for the presence of the plasmid backbone using the insertion specific AP3/AP4 PCR, and successful excision was confirmed (Figures 7D,E). On the other hand, as we expected, PBase expression successfully excised only the *Puro∆TK* cassette from Ic14 genomic DNA, leaving the plasmid DNA backbone and exon 23 tandem duplication intact (Figures 7D,E). Finally, to confirm generation of a seamlessly corrected *W1282X*-wt allele through IHR, we sequenced all 12 recombinant clones and confirmed the A > G correction and absence of NHEJ products. (Figures 7E,F, representative sequences from clones 34, 35 and 36). Similar results were obtained in an independent trial followed by negative selection with FIAU (Supplemental Materials: Supplementary Figure S9). These data suggest that successful excision via IHR can provide seamless corrected clones. We confirmed normal pluripotent stem cell characteristics in clones Ic14_GCV_1.2-c that had successfully undergone HDR with additional donor, and Ic14_FIAU_c-b10.9 and Ic14_GCV_c-e36 that had IHR without additional donor. All three clones with excised insertions by both HDR and IHR expressed the pluripotent markers in iPSCs culture condition and three germ layer markers in the randomly differentiated cultures (Supplemental Materials: Supplementary Figure S10A). All the vector-insertion removed CF2-iPS3 cells had normal male karyotypes (Supplemental Materials: Supplementary Figure S10B). Thus, we demonstrate a novel and effective strategy to obtain seamless gene correction by IHR of vector insertion products.

## Discussion

Here we demonstrate two approaches that can lead to making seamless gene corrections in CF-iPSCs homozygous for the *W1282X* mutation. One approach uses established vector replacement HDR events catalyzed by CRISPR/Cas9n generated DSBs followed by treatment with the PiggyBac transposase system to remove drug selectable markers. We also demonstrate a novel strategy using cell lines containing site-specific, homologous recombinant, vector insertion events followed by excision of drug selectable markers and duplicated DNA sequences *via* CRISPR/Cas9n stimulated intrachromosomal recombination, similar to I-SceI endonuclease stimulated IHR (Sargent et al., 1997; Donoho et al., 1998; Liang et al., 1998; Sargent et al., 2000).

The relative frequency of CRISPR/Cas9n stimulated vector replacement events ranged from 5.7 to 28.6% homologous recombinants per puromycin resistant colony. This frequency appears to be dependent on the amount of homology in the vector arms with the more extensive homology correlated with the higher relative recombination frequency. Whereas increasing the total amount of vector homology approximately 1.7-fold, from 1,370 bp (vector CF2A, Figures 2A,B) to 2,355 bp (vector CF2B, Figures 2A,B), we observed an approximate 5-fold increase in vector replacement products (Figure 2B). This result is similar to observations using classical gene targeting in mouse embryonic stem cells (without CRISPR/Cas9) where increasing the total homology present in targeting vectors from 1.3 to 6.8 kbp resulted in approximately a 250-fold increase in homologous recombination (Hasty et al., 1991). The experiments presented here are from individual experiments, thus future investigations to quantify the influence of homology between targeting vectors and genomic targets, and how that homology is distributed, will require more experimentation with multiple biological replicates.

Unlike previous gene targeting experiments in mouse ES cells using vectors linearized inside homologous vector arms, or at the border of arm homology and vector sequences (Figure 1) (Valancius and Smithies, 1991; Hasty et al., 1994), we co-transfected the CF2-iPS3 cells with unlinearized circular targeting vectors and CRISPR/Cas9n with gRNA designed to target or preferentially cut the *W1282X-*mutant genomic DNA sequence. While this approach was used to minimize cutting in vector-exon 23 sequence, we cannot rule out that the vector replacement and vector insertion events observed here were due to some CRISPR/Cas9-pair-2 nicking of vector DNA or possibly nonspecific nicking of vector DNA by cellular enzymes or during the nucleofection process. However, if HDR is initiated between a chromosomal break and a circular targeting vector, then the double-strand-break-repair (DSBR) and Holliday models for homologous recombination predict two possible HDR products: vector replacement events and vector insertion events (Holliday, 1964, 1968; Szostak et al., 1983). In support of this prediction, we observed vector insertion events for both the CF2A and CF2B targeting vectors from independent experiments (Figure 4).

The majority of vector insertion products are homologous recombinants with only one product out of seven sequenced clones (one out of 14 junctions) that showed evidence of an accompanying sequence duplication (Lc35.11; Figure 4B), unlike the strategy of whole plasmid DNA insertion resulting in a high rate of indel inductions within junctions *via* NHEJ (Maresca et al., 2013; He et al., 2016). Interestingly, the majority of clones with HDR vector insertion products also had *W1282X* A-to-G gene conversion events such that both of the duplicated exon 23 sequences contained the *WT* CFTR gene sequence. The TAA sequence duplication in the Lc35.11 clone could be explained by mis-templating of DNA synthesis on the vector DNA, accompanying DSBR, that would also be responsible for incorporating the observed A to G reversion. The *W1282X* gene conversion events could be caused by DNA strand recession at the genomic exon 23 target and repair by DNA synthesis or possibly through heteroduplex formation between vector and genomic sequences followed by DNA repair of mismatched sequences between the genomic *W1282X*-mutant and vector *W1282X*-wt sequences. Future experiments with multiply marked genomic and vector DNA sequences may help to distinguish between these two models to explain the gene conversion events associated with vector insertion events. While these studies have demonstrated correction of *W1282X*-mutant DNA sequences, demonstration that the corrected *W1282X*-wt allele is truly functional will require differentiation of the iPS cells into products that express the corrected full length CFTR protein and the truncated W1282X protein for detection and demonstrate chloride channel activity from the full length CFTR protein.

We observed a relative HDR vector insertion frequency of 57.1% for the CF2A vector, whereas there was only a 19% frequency of HDR vector insertion using the CF2B vector. For the CF2A vector, this represents an approximate 10-fold higher frequency of insertion products to replacement products within the same puromycin resistant cell population. The ratio of HDR vector insertion events as compared to replacement events appears to be inversely dependent on the length of homology present in vector arms since the ratio of insertion to replacement products decreases from 10:1 to 1:1.5 (Figure 4). This result is similar to what has been observed in classical gene editing approaches where homology arm length had a direct effect on increasing vector insertion gene targeting efficiencies (Hasty et al., 1991). Indeed, experiments in mouse ES cells demonstrated that gene targeting efficiencies for insertion vectors were nine-fold better than replacement vectors with same homology arm lengths (Hasty et al., 1991b). In those experiments linearized targeting vectors were electroporated into mouse ES cells without genomic DSBs. Our experiments were designed to minimize or prevent vector cutting by the CRISPR/Cas9n proteins hence the ratio of vector replacement versus vector insertion events reported here may reflect HDR products as initiated from genomic DSBs. It will be interesting to further test the relationship between plasmid homology arm length and vector replacement versus vector insertion events at different gene targets. If the HDR process leading to vector insertion events is dependent on DSB formation then we predict that we should observe similar results using purified Cas9n protein as ribonucleotide protein complexes. One advantage of the Cas9 RNP approach is the potential for fewer off target events from reduced persistence of active Cas9 as compared with plasmid based Cas9 expression (Kim et al., 2014; Lin et al., 2014; DeWitt et al., 2017).

Excision of the *Puro∆TK* drug resistance cassette from the lc8 cell line, containing a vector replacement product, was accomplished by transfecting cells with a PB transposase expression vector and selecting for GCV or FIAU resistant colonies. Both drug selection protocols yielded approximately the same relative frequency of cell lines with the *Puro∆TK* drug resistance cassette removed (1/7 versus 1/6) as determined by PCR analysis. DNA sequencing confirmed the CF2-iPS3_FIAU_e11 clone contained a seamless excision of the *Puro∆TK* drug resistance cassette whereas the CF2-iPS3_GCV_e2 clone also contained an 11 basepair deletion in the untargeted CFTR exon 23, adjacent to CRISPR/Cas9n pair2 targeting site and to the same TAA sequence found duplicated in the insertion product Lc35.11 clone. Both clonal lines had normal diploid karyotypes, showed expression for pluripotent markers, and could be differentiated into cells from the 3 germ layers (Supplemental Materials Supplementary Figure S4).

Two strategies were used to stimulate recombination between the exon 23 duplication in the lc14 cell line containing a vector insertion product. The first approach used the CRISPR/Cas9n-pair-2w nickases that could cut in either or both copies of exon 23 in the duplicated locus. If both copies of exon 23 received double strand breaks, then the resulting intermediate would be repaired by NHEJ and if only one copy is cut the resulting product could be repaired by HDR or possibly by NHEJ. To reduce the possibility of NHEJ, we performed experiments where a short DNA fragment (SDF) homologous to the *CFTR* exon 23 was cotransfected to provide an exogenous substrate for repair of the double strand breaks induced by the CRISPR/Cas9-pair-2w nickases. Cotransfection of the CFTR SDF did not appear to dramatically improve recovery of recombinant cell lines and may have interfered with recovery of GCV^R^ colonies (Figure 6B). However, in these experiments, 1/5 GCV resistant clones were homologous recombinants versus 1/12 (Figure 6B) or 0/8 homologous recombinants without the SDF (Figure 6B). We are conducting experiments to further quantify the influence of an uncleavable exogenous template for CRISPR/Cas9 stimulated DSBR involving insertion products with tandem gene duplications.

The second approach to stimulate IHR between the exon 23 duplications tested the ability of double strand breaks near the *CFTR* homology, but inside the vector backbone, or at the edge of the vector/*CFTR* sequences to induce recombination. Transfection of the lc14 cell line with the CRISPR/Cas9n-pair-M13T7/5′CF2 stimulated appearance of GCV^R^ colonies at a frequency similar to transfection with the CRISPR/Cas9n-pair-2w or with transfection of the PBase transpose expression vector. However, for cells treated with the CRISPR/Cas9n-pair-M13T7/5′CF2, 12/12 gancyclovir recombinants were confirmed as seamless homologous products that reconstructed a gene corrected *W1282X*-wt sequence (Figure 7F, clones c-e34, e35 and e36 are shown as representatives). With cells treated with the CRISPR/Cas9-pair-2w nickase, 3/3 gancyclovir resistant clones were due to NHEJ and deleted all or part of exon 23 (Figure 7F, clones a-e57, e61 and e97).

**FIGURE 8:**
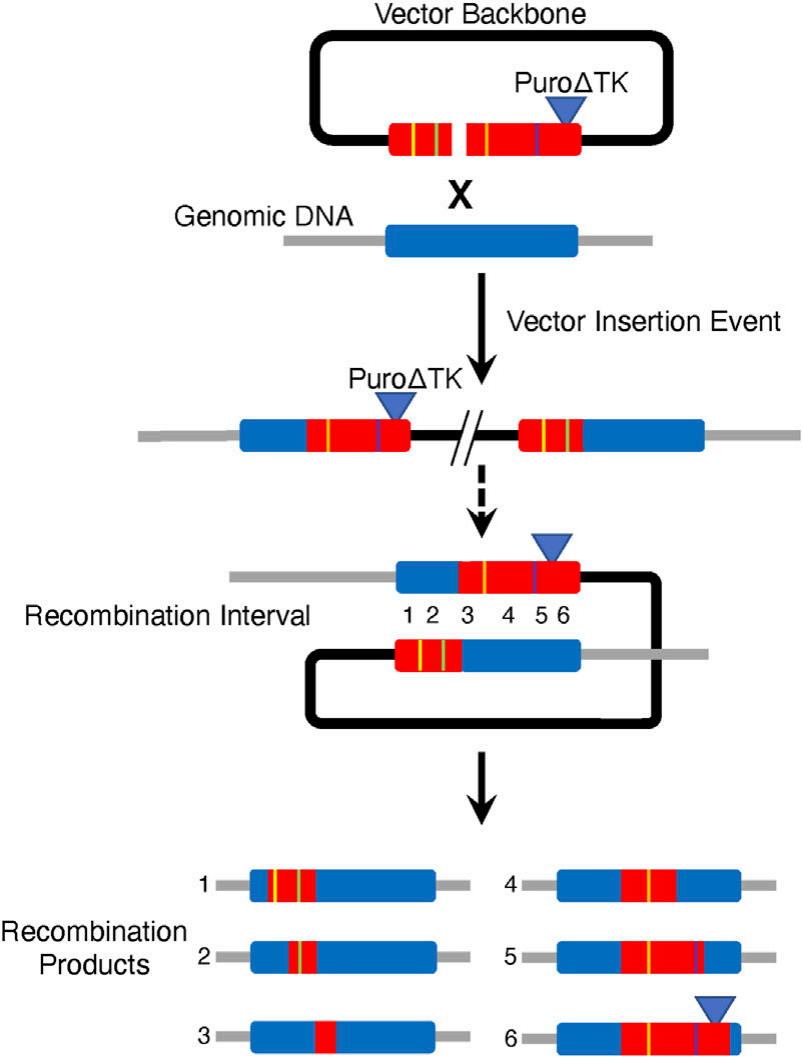
Strategy for Producing Multiple Recombinant Cell Lines from One Parental Vector Insertion Event Cell Line. Generation of multiple cell lines each containing different combinations of mutations could be generated from one parental vector insertion cell line using a multiply mutant targeting vector. In this example, product cell lines would generated by IHR across different recombination intervals.

Generation of homologous recombinant cell lines from vector insertion products has several potential advantages to create many genetically different progeny cell lines from one gene targeting event (Figure 8). This study demonstrates the potential to create seamless gene corrections for cell lines in the same number of steps as commonly used strategies involving the PiggyBack transpose. By creating double strand breaks at the border of vector sequences and homologous genomic sequence we were able to stimulate HDR between tandemly duplicated *CFTR* exon 23 and propose that CRISPR/Cas9 M13T7-based nickases could serve as almost universal nickases when paired with a target-specific genomic DNA nickase to stimulate IHR in vector insertion products. We also suggest that one could engineer cell lines with vector insertion products using vectors containing multiple gene sequence changes. Recombination in the intervals between the sequence changes, induced by a CRISPR/Cas9 targeted to generate DSBs in the interval, would then result in cell lines containing the desired mutation(s) (Figure 8).

Many laboratories appear to focus on screening for only vector replacement events when using CRISPR/Cas, TALEN, and ZFN proteins to stimulate HDR and engineer genetic changes or knockins in genomic DNA. The frequent isolation of vector insertion events observed in these studies suggest that when using circular donor DNA templates with CRISPR/Cas nucleases that a large number of discarded or unrecognized recombination products might be useful vector insertion events. Indeed, we have observed similar results consistent vector insertion events using CRISPR/Cas9 with unlinearized, circular plasmid donor DNA vectors for HDR in human iPS cell lines at the hemoglobin subunit beta (HBB) locus and the glial cells missing transcription factor 2 (GCM2) gene (unpublished observations). Further experiments will investigate the influence of homology arm length on the ratio of vector insertion/vector replacement events using plasmid expressed CRISPR/Cas9 and CRISPR/Cas9 purified proteins.

## Materials and Methods

### Cells and Culture Conditions

All studies that involve human iPSCs were approved by the UCSF Human Gamete, Embryo and Stem Cell Research (GESCR) Committee. Immortalized CFBE41o- cells are homozygous for the *F508del CFTR* mutation (Gruenert et al., 2004; Illek et al., 2008) and were routinely grown in supplemented Eagle’s Minimal Essential Medium and subcultured with PET [0.02% trypsin-versene (UCSF CCF), 1% polyvinylpyrrolidone (Sigma-Aldrich), and 0.2% EGTA (Sigma-Aldrich)] as described previously (Shingo Suzuki et al., 2016). CFBE41o- cells were transfected with CRISPR/Cas9n-gRNA pairs to test for nuclease activity using the T7 endonuclease I assay (New England Biolabs, T7E1 assay, see Materials and Methods in Supplemental Materials for more detail). CF2-iPSCs, homozygous for the *W1282X* G > A mutation were provided by Dr. Gruenert at UCSF and routinely cultured on Matrigel (BD Biosciences, San Jose, CA) in mTeSR1 medium (StemCells Inc., Vancouver, BC, Canada) and subcultured by non-enzymatic dissociation with ReLeSR (StemCell Inc.).

Cell passage number is denoted as Pn_1_.n_2_.n_3_.—n_z,_ where n_1_ = number of passages as primary cells before reprogramming, n_2_ = number of passages since reprogramming, n_3_ = number of passages after transfection with a donor DNA, etc., where each period delineates the onset of a specific protocol or treatment that alters the character of the cells (Gruenert et al., 1988).

### Generation of Embryoid Bodies

CF-iPSCs lines were cultured in mTeSR1 medium on Matrigel, and then harvested by treatment with Dispase, followed by centrifugation (5 min at 200 g). Cell pellets were re-suspended in 2 ml of mTeSR1 supplemented with 10 μM Y27632 (Selleckchem, Houston, TX), re-plated into 1 well of a 6-well low attachment tissue culture plate (Corning-Costar) and cultured at 37°C under 5% CO_2_. Within 24 h, floating 3-dimensional spherical cell clumps indicative of Embryoid Bodies (EBs) were visible. EBs were cultured for additional 7 days in mTeSR1 with feeding every other day and then transferred to a multi-well tissue culture plate coated with 0.1% gelatin for attachment. The cells were then grown for an additional 7 days in DMEM supplemented with 10% FBS, 2 mM l-Glutamine, 1 × Non-essential amino acid (UCSF CCF), and 1 × PenStrep before immunostaining.

### Immunocytochemical Analysis

Cells were grown in multi-well plates on Matrigel (iPSCs) or on gelatin-coated surfaces (non-iPSCs) for immunostaining using primary antibodies and fluorescently labeled secondary antibodies (Supplemental Materials: Supplementary Table S1). Briefly, cells washed with PBS were fixed in 4% PFA for 30 min at room temperature (RT) and washed three times, for 5 min each, with ice-cold PBS. Samples were then permeabilized with 0.25% Triton-X for 10 min at RT and incubated for 45 min with 5% serum and 1% BSA in PBS containing 0.1% Tween20 (PBST) at RT with gentle agitation, followed by an overnight incubation at 4°C with primary antibody in 1% BSA in PBST. Samples were then subjected to 3 × 5 min washes with PBS by gentle agitation. After the third wash, the cells were incubated with secondary antibody in 1% BSA, PBST for 1 h at RT in the dark, and again washed 3 times as indicated above. Samples were sealed with Dapi Fluoromount-G (SouthernBiotech, Birmingham, AL) and coverslips and examined by fluorescence microscopy.

### Cytogenetic Analysis

CF-iPSCs were treated with 10 ng/ml of Colcemid (Invitrogen) at 37°C for 1 h. The cells were harvested and G-banded according to standard cytogenetic protocols (Barch et al., 1997). Metaphase cells were analyzed and karyotyped with the CytoVision system (Leica Microsystems, Wetzlar, Germany) by the Department of Laboratory Medicine Cytogenetics Core, UCSF. Chromosomes were also stained using Quinacrine (Sigma Aldrich) for 30 min incubation and karyotype analysis was made through Genikon software (Nikon, Tokyo, Japan) by Department of Molecular Medicine, University of Pavia.

### PCR

Genomic DNA was isolated with GeneJET Genomic DNA Purification Kit (Thermo Fisher scientific, Waltham, MA) and used for PCR reactions. PCR were performed using 2X MyTaq HS Mix (BIOLINE, Tuanton, MA) or PrimeSTAR GXL DNA Polymerase (Takara Bio USA Inc., Mountain View, CA) according to the manufacturer’s instructions. The amplification products were separated by 0.8-2% agarose electrophoresis, depending on product size, and imaged with UV light on a Geldoc 2000 imaging instrument (Bio-Rad, Hercules, CA). PCR primers are indicated in Additional file 1: Supplementary Table S2.

### Donor DNA Vector Construction

The *Puro∆TK* expression cassette was derived from pCAG-PuroΔTK.Neo (Ye et al., 2014) by removing the Neomycin gene. Two *CFTR* exon 23-targeting vectors (CFTRexon 23-pCAG-PuroΔTK. Neo; CF2A or CF2B) were constructed using recombinant PCR. Briefly, CF2A consists of 804-bp of the 5′ homology *CFTR* arm containing the intron sequences upstream of *CFTR* exon 23 (CF2A-2 fw, 5′- TTG​CAG​GTC​TCT​GCT​TCT​GG -3′) through the first TTAA site from exon 23 in the intron downstream of exon 23 (TGTTTTTTAA). The 3′ homology arm consists of 566-bp from the intron TTAA site above (TTAACAGCTC) to GAGCACCTCC (CF2A rv, 5′- GGA​GGT​GCT​CCT​GGC​ATT​TTA -3′) in the same intron. CF2B consists of 1207-bp of the 5′ homology *CFTR* arm containing AGAACACAGA (CF2B fw, 5′- AGA​ACA​CAG​AGT​TGG​GGC​TC -3′) through the same TTAA site as CF2A construct. The 3′ homology arm consists of 1148-bp from the intron TTAA site to GGCCAGAGTT (CF2B rv, 5′- AAC​TCT​GGC​CCA​CTT​GGT​TTT -3′) in the same intron. These homology arms were amplified by PCR and joined with the CAG-PuroΔTK cassette by recombinant PCR to create the final targeting construct. All primers used for donor DNA construction are shown in Supplemental Materials: Supplementary Table S3.

### Generation of CRISPR/Cas9n-gRNAs

Guide RNA targeting sequences were designed and selected using Web-based software, Optimized CRISPR design, developed by Zhang Lab, MIT. pSpCas9n (BB)-2A-Puro (PX462), a gift from Dr. Feng Zhang (Addgene plasmid # 48,141), was used as the both gRNA and SP-Cas9n expression vector. The oligos listed in Supplemental Materials: Supplementary Table S4 were used to assemble gRNA targeting specific sequence by following the established protocol (Ran et al., 2013b).

### Correction of *W1282X* Mutation in CF2-iPS3 Cells

CF2-iPS3 cells were co-transfected with the donor DNA plasmid (CF2A or CF2B) in the absence or presence of CRISPR/Cas9n-gRNAs. Briefly, CF2-iPS3 cells were harvested as single cell suspension with Accutase (StemCell Inc.). Then, 1.5 × 10^6^ cells were nucleofected with 2.5 μg of donor DNA plasmid with or without 2.5 μg of each pairs of CRISPR/Cas9n-gRNAs using the 4D Nucleofector X (Lonza) with the P3 Primary Cell solution and Program CA137. Transfected CF2-iPS3 cells were plated in mTeSR1 supplemented with 10 μM Y27632 in Matrigel-coated plate for 24 h post transfection. Two to 3 days after transfection, the culture medium was switched to the selection medium, mTeSR1 containing 0.5–1.0 μg/ml of Puromycin (Sigma Aldrich), and the cells were continuously cultured under Puromycin selection up to 14 days. During the selection, 7–10 days post-transfection, all colonies were manually picked and individually transferred to 24-well plates coated with Matrigel. Genomic DNA was isolated from individual clones and amplified by PCR with primers AP1/AP2, AP3/AP4, and P6r/P7f to screen for successful vector replacement and insertion events, and each PCR product was separated on a 1-2% agarose gel containing ethidium bromide and visualized under UV light.

### Excision of Puro∆TK Cassette

In order to remove the *Puro∆TK* cassette containing the drug selection markers from modified CF2-iPS3, a PiggyBac transposase (PBase) expression vector was transfected into recombinant cells, followed by negative selection with Fialuridine (FIAU) (Santa Cruz Biotechnology Inc., Dallas, TX) or Ganciclovir (GCV) (Sigma-Aldrich) (Chen and Bradley, 2000). The modified PBase (R372A/K375A/D450N) (Li et al., 2013) expression vector was kindly provided by Drs. YW Kan and Lin Ye at UCSF. The PBase expression vector was nucleofected as described above. After nucleofection, the cells were passaged twice as single cells every 2–3 days with Y27632, and plated into 60 mm Petri dishes, as single cells, at 10^6^ cells/dish. Negative selection for loss of the TK gene was with mTeSR1 medium containing 0.25 μM FIAU, or 0.2 or 2 μM GCV. After negative selection, colonies were clonally isolated and cultured individually in 24-well plates. PCR amplification with primers P8/P4, P6r/P7f were performed on genomic DNA harvested from each clone to screen for successfully excised *Puro∆TK* cassette (map with PCR primer locations). The percentage of excision was calculated from the number of *Puro∆TK* cassette negative clones (P8/P4^-^ and P6r/P7f^−^) out of the cell numbers right before the selection in 60 mm dish (10^6^ cells), according to the following formulas:

$$f_{\mathrm{excision}}=\frac{\mathrm{Excised}\;\mathrm{clones}}{\mathrm{tested}\;\mathrm{clones}}$$

%Excision=100∗All appeared colonies∗fexcision10^6

### Excision of Whole Plasmid Vector Insertions

CRISPR/Cas9n targeting *CFTR* exon 23 or the junction of vector backbone and CFTR gene were nucleofected into CF2-iPSCs (Ic14) to introduce Intrachromosomal HDR. After nucleofection, excised clones were screened with 0.2 or 2 μM GCV, or 0.25 μM FIAU and analyzed by PCR for excision of the *Puro∆TK* cassette as described above. The percentage of excision was calculated as described above. The analysis of seamlessly excised clones was performed by PCR with the CF46/CF47 PCR primer pair and then confirmed by Sanger sequencing on AP1/4 PCR amplicons.

## Data Availability

The original contributions presented in the study are included in the article/Supplementary Material, further inquiries can be directed to the corresponding authors.
