# Supplementary material for: Seamless Gene Correction in the Human Cystic Fibrosis Transmembrane Conductance Regulator Locus by Vector Replacement and Vector Insertion Events
Source: Front Genome Ed. 2022 Apr 6;4:843885. doi: 10.3389/fgeed.2022.843885 (PMC9019469; doi:10.3389/fgeed.2022.843885)
Supplement: Supplementary file 1 [file DataSheet1.pdf]

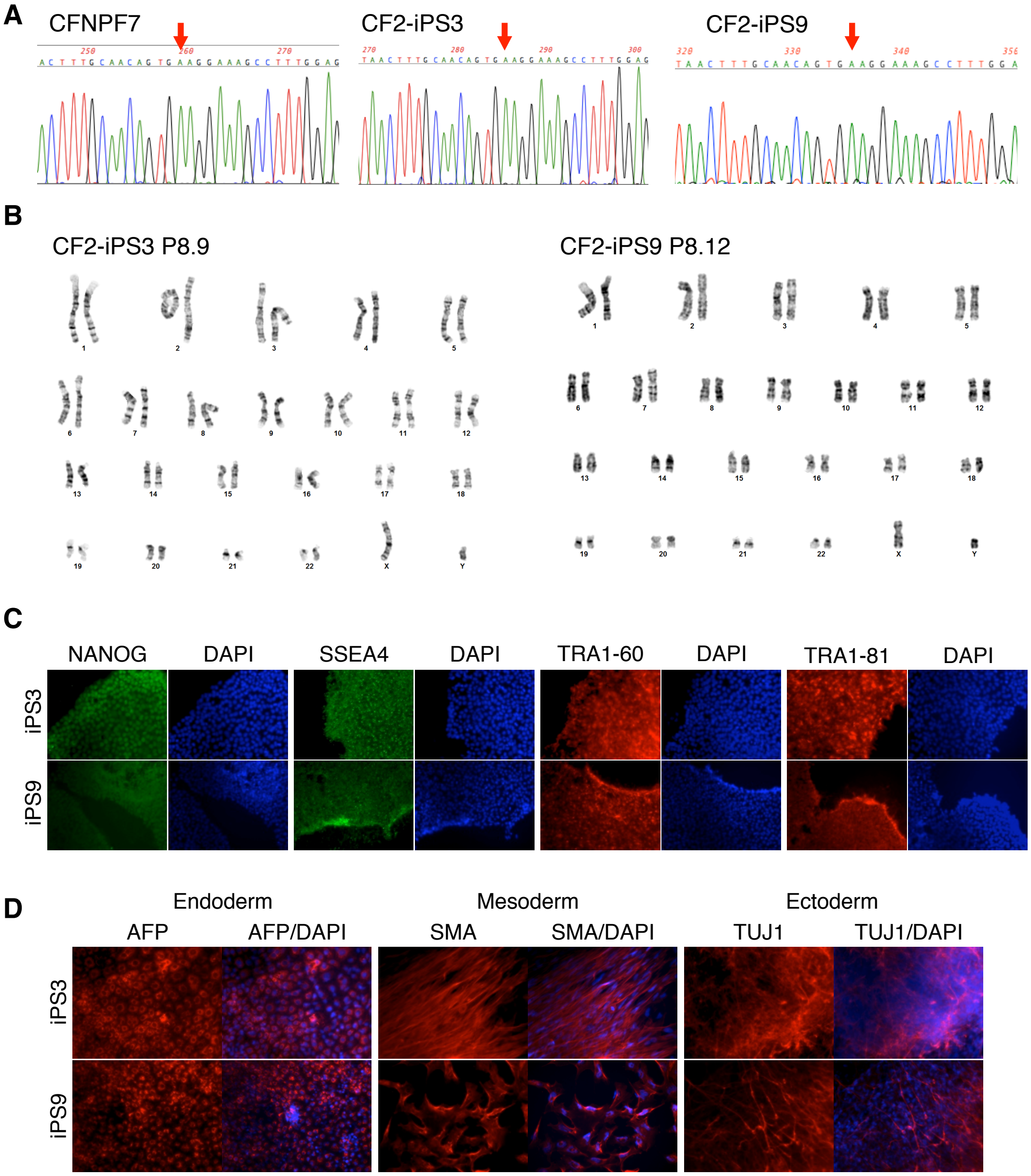

Supplemental Fig S1 Generation of *W1282X* CF2-iPSCs. (A) Genetic characterization of parental fibroblast (CFNPF7) and reprogrammed CF-iPSCs (CF2-iPS3 and –iPS9 cells) by Sanger sequencing (B - D) Characterization of CF2-iPS3 and -iPS9 cells by karyotyping (B) and immunocytochemical analysis for ES (C) and three-germ layers markers (D).

A

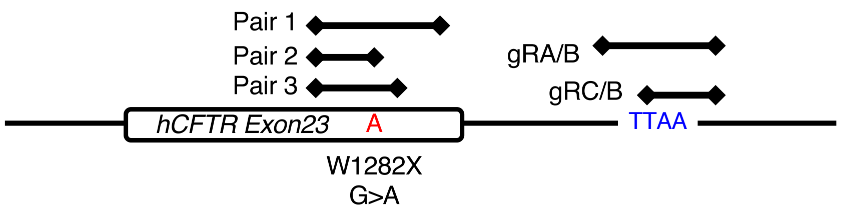

CRISPR Cas9n	5' gRNA (5'>3')	Target mutation	3' gRNA (5'>3')	Target mutation	gRNA offset
Pair 1	CCCAAGACACACCATCGATC tgg	No	AGCCTTTGGAGTGATACCAC agg	No	28-bp
Pair 2			CAATAACTTTGCAACAGTGA agg	Yes	3-bp
Pair 3			CAACAGTGAAGGAAAGCCTT tgg	Yes	14-bp
Pair 2w			CAATAACTTTGCAACAGTGG agg	No	3-bp

gR	5' gRNA (5'>3')	gR	3' gRNA (5'>3')	offset
A	CAAGGCTCCCACTGTAAATT tgg	B	CTTTTTTGTTCCTGCTTCAG tgg	21-bp
C	TGTTAAAAAACAAAAGCCCA agg			3-bp

B

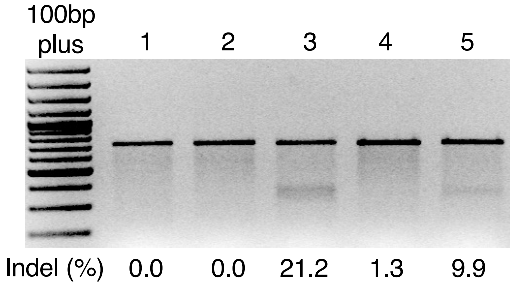

lane	Nuclease pair	Fragment size (bp)	
1	Mock	709	
2	5' gR alone	709	
3	gR Pair 1	357	352
4	gR Pair 2	345	364
5	gR Pair 3	350	359

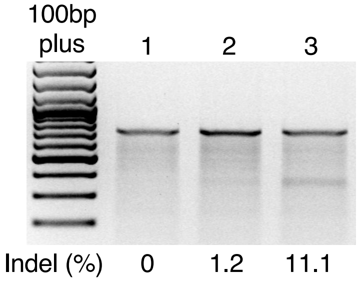

lane	Nuclease pair	Fragment size (bp)	
1	Mock	709	
2	gR Pair 2	345	364
3	gR Pair 2w	345	364

Supplemental Fig S2 Assessment of CRISPR/Cas9n-gRNAs for *W1282X* targeting. (A) Targeting sites and sequence of CRISPR/Cas9n-gRNAs used for *W1282X CFTR* targeting. Each gRNA targeting site is represented by black diamond and connected by the line for a pair. G>A mutation causing *W1282X* mutation is highlighted in red and also TTAA site used to insert PB cassette is colored in blue. (B) T7E1 assay for exon23 targeting CRISPR/Cas9n-gRNAs. %Indel is shown under the gel picture (left panels) corresponding each pairs of CRISPR/Cas9n-gRNAs listed in the table with digested fragment sizes (right tables).

A

Experiment	Cell numbers	donor DNA (µg)	CRISPR (µg each)	Puro (µg/ml)	Puro^R	HDR	Insertion
H	1.5 x10^6	CF2A (2.5)	-	0.5	28	0	0
I	1.5 x10^6	CF2A (2.5)	Pair 2 (2.5)	0.5	35	2 (5.7%)	20 (57.1%)
J	1.5 x10^6	CF2A (2.5)	C/B (2.5)	0.5	36	0	0
K	1.5 x10^6	CF2B (2.5)	-	0.5	31	0	0
L	1.5 x10^6	CF2B (2.5)	Pair 2 (2.5)	0.5	42	12 (28.6%)	8 (19.0%)
M	1.5 x10^6	CF2B (2.5)	C/B (2.5)	0.5	32	0	0

B

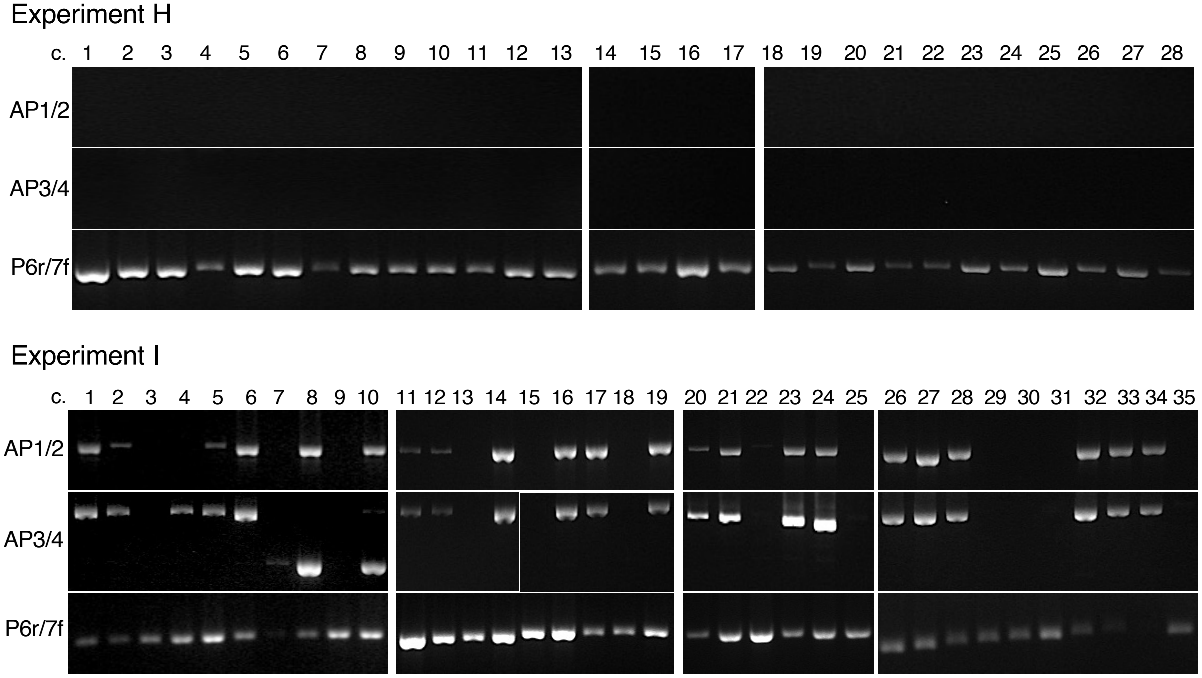

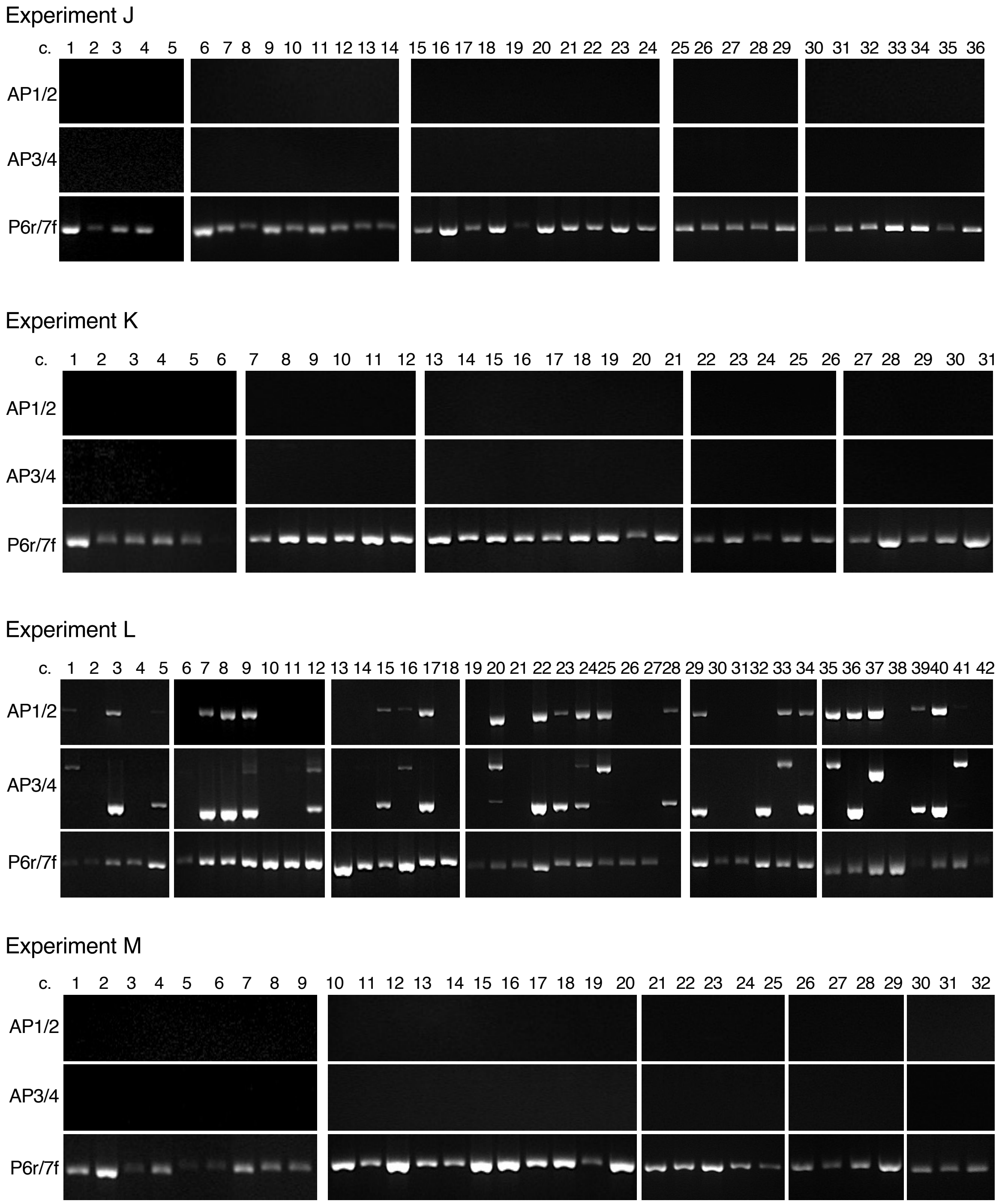

Supplemental Fig S3 All PCR pictures for 1st screening of Puro^{R} clones. (A) The table showes all experimental information for 1st screening. (B) Gel pictures of genotyping for all colonies during selection for CF2-iPS3 cells that are transfected with donor DNA with and without CRISPR/Cas9n-gRNAs and then are treated with 0.5 µg/ml Puromycin. Genomic DNA were isolated from individual clones and amplified by PCR with primers AP1/AP2, AP3/AP4, and P6r/P7f to screen successful vector replacement and insertion event, and each PCR product was separated on a 1 - 2% agarose gel.

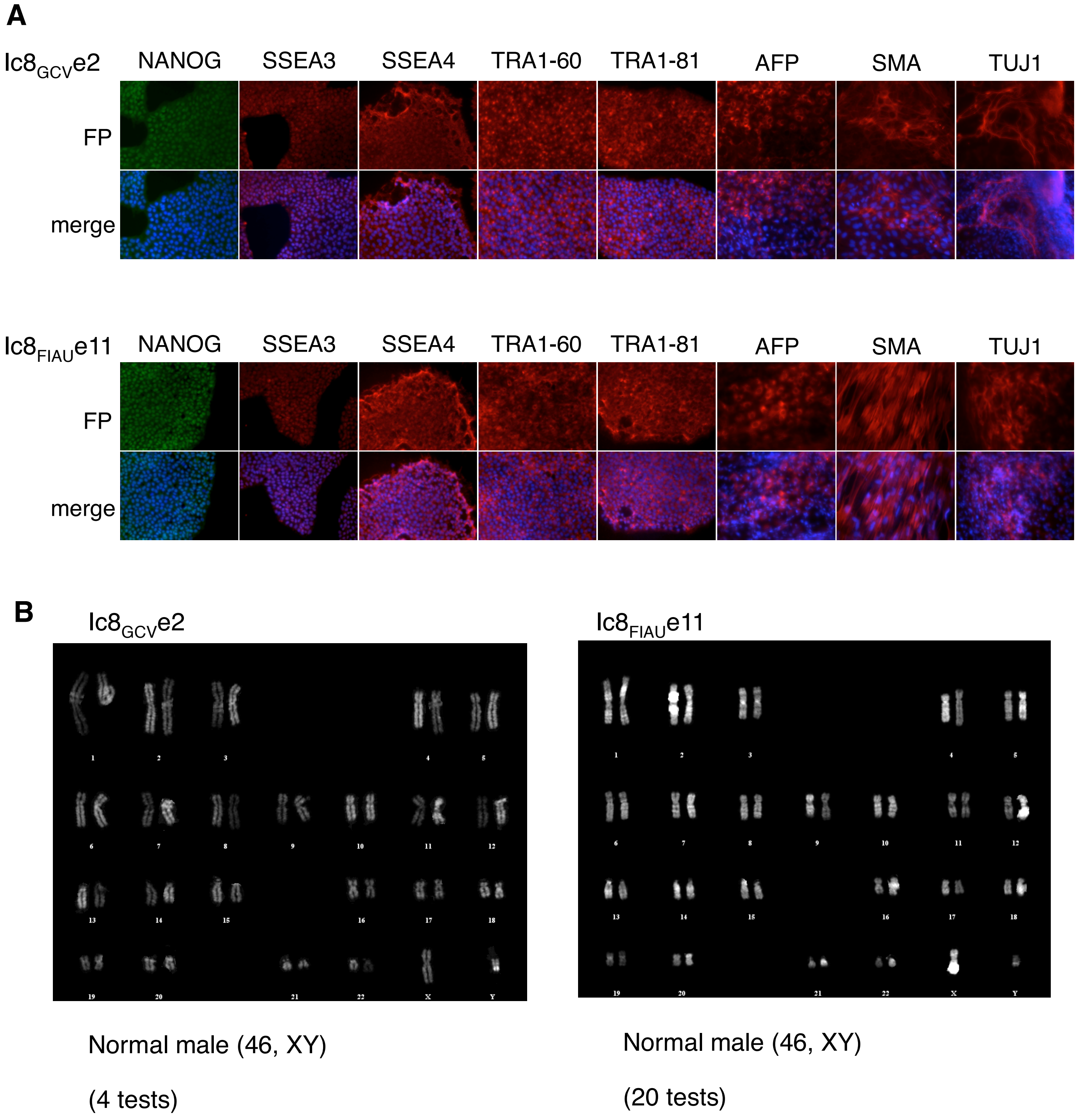

Supplemental Fig S4. Characterization of corrected Class I CF mutation in CF2-iPS3 cells. (A, B) Characterization of Ic8GCVe2 and Ic8FIAUe11 clones by (A) immunocytochemical analysis for ES markers (NANOG, SSEA3, SSEA4, TRA1-60, TRA1-81), and three-germ layers (Endoderm: alpha-fetoprotein; AFP, Mesoderm: alpha-smooth muscle actin; SMA, Ectoderm: beta-tubulin 3; TUJ1), and by (B) karyotype.

A

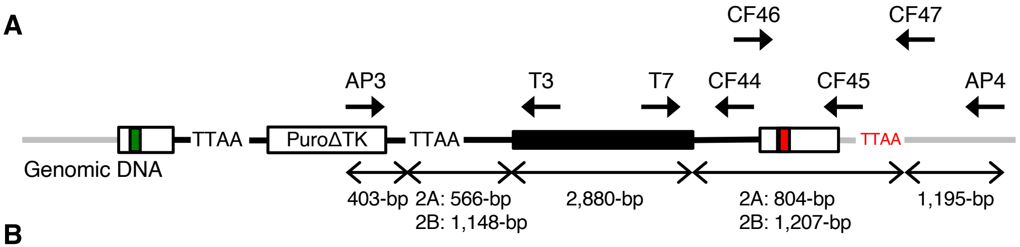

B

Donor DNA (5': bp/ 3': bp)	Primer pair (F/R) and product size (bp)				
	AP3/AP4	AP3/CF44	AP3/T3	CF46/CF47	T7/CF45
CF2A (804/566)	5,848	4,003	1,018	709	583
CF2B (1207/1148)	6,833	4,988	1,600	709	986

C

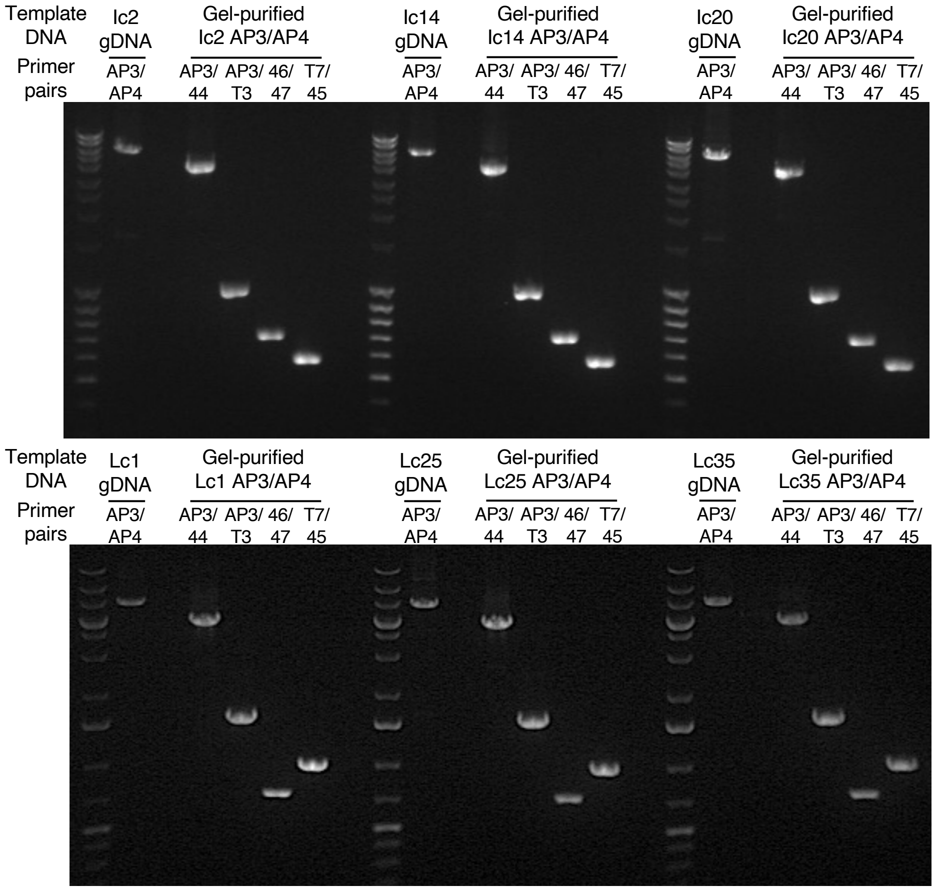

Supplemental Fig S5 Identification of HMW by PCR experiments. (A) Schematic illustration for identification of HMW band by PCR with primer pairs AP3/AP4, AP3/CF44, AP3/T3, CF46/CF47, and T7/CF45. (B) Primer pair and predicted product size obtained from each PCR experiments on CF2A or CF2B donor DNA-transfected CF2-iPS3 cells. (C) Gel pictures of genotyping for representative colonies by PCR. Upper; clones transfected with CF2A donor DNA. Bottom; clones transfected with CF2B donor DNA.

A

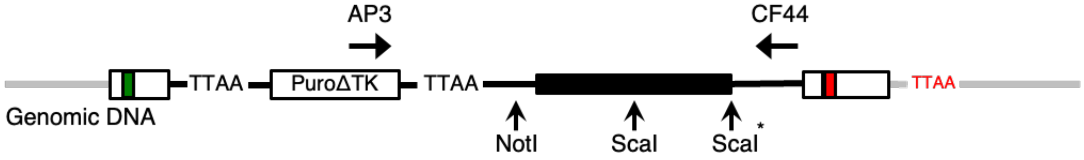

B

Donor DNA (5': bp/ 3': bp)	Restriction Enzyme and Fragment size (bp)			
	-	NotI	ScaI	NotI/ScaI
CF2A (804/566)	4,003	3,030 973	2,753 1,250	1,780 1,250 973
CF2B (1207/1148)	4,988	3,433 1,555	3,335 1,308 345	1,780 1,555 1,308 345

C

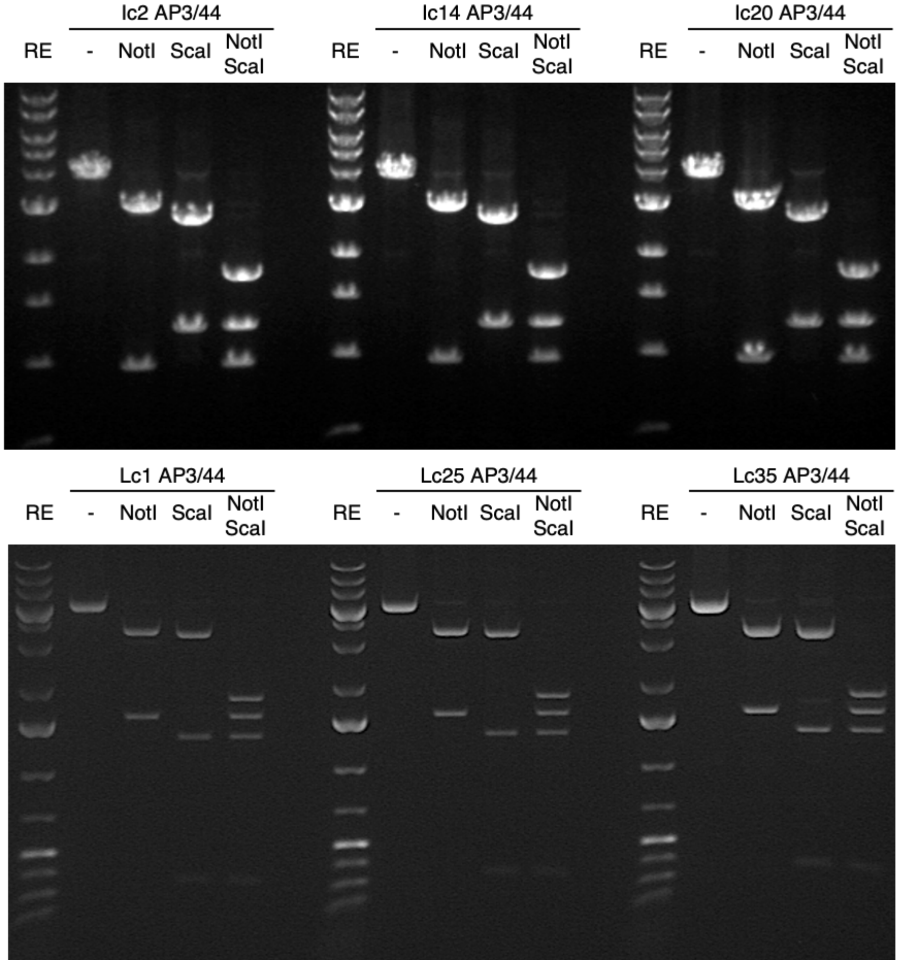

Supplemental Fig S6 Identification of HMW by restriction enzyme (RE) digestion. (A) Schematic illustration of HMW bands by RE cutting of PCR products. PCR with primer AP3/CF44 was performed on genomic DNA harvested from each clone. These PCR products were gel-purified and digested by NotI and/or ScaI. *ScaI cutting site next to the CF44 primer annealing site exists only in CF2B donor DNA. (B) RE and predicted fragment size obtained form each RE digestion experiment on CF2A or CF2B donor DNA-transfected CF2-iPS3 cells. (C) Gel pictures of RE digested fragments for representative colonies. Top; clones transfected with CF2A donor DNA. Bottom; clones transfected with CF2B donor DNA.

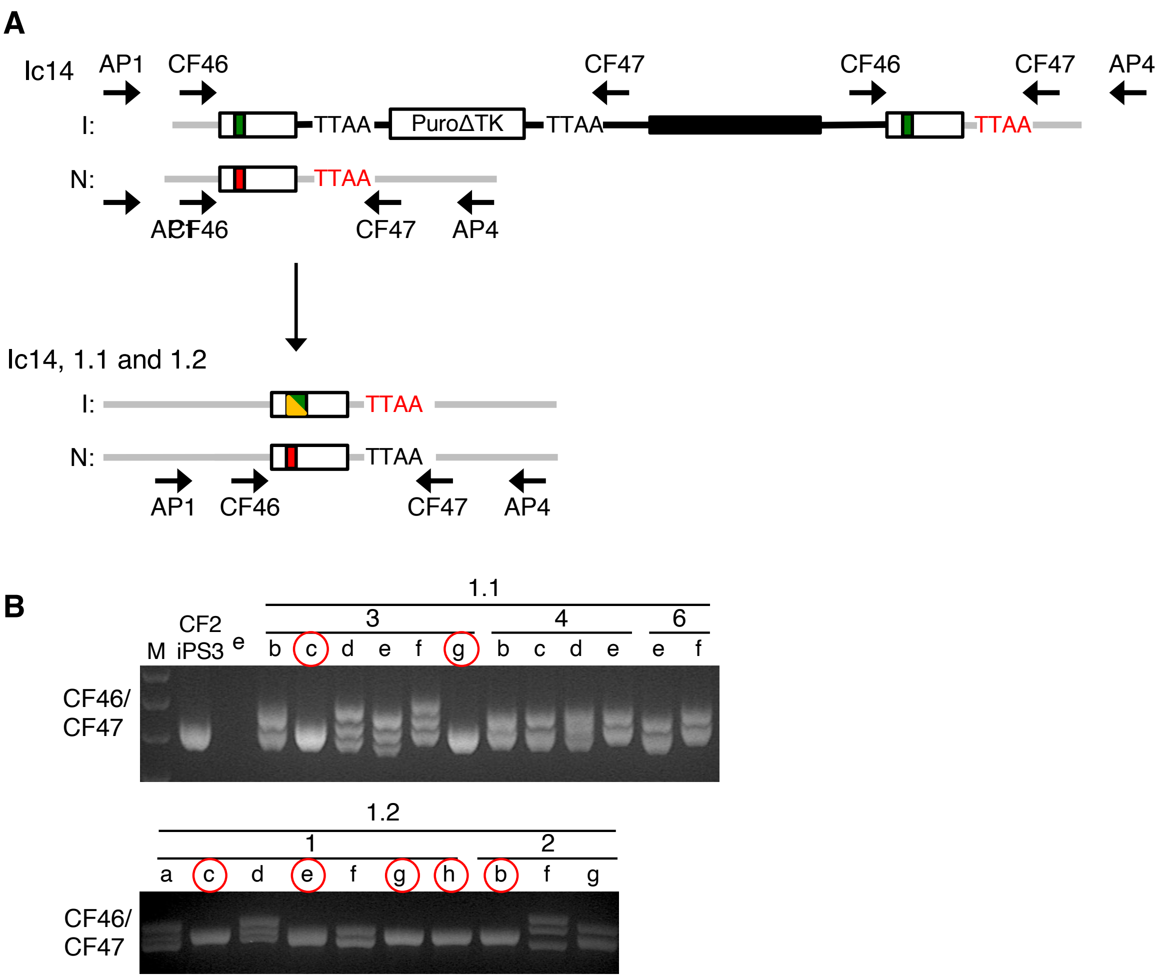

Supplemental Fig S7 Screening for seamless excision. (A) Schematic illustration of excising inserted backbone in order to obtain seamless corrected CF2-iPS3 cells using CRISPR/Cas9n-pair 2w with or without SDFs. (B) Selection of possible seamless excised clones with CF46/47 PCR. PB cassette negative clones from treatment 1.1 and 1.2 in Fig 4 were tested in a multiplicity of amplicon. Clones 1.1-3c and -3g, and 1.2-1c, -1e, -1g, -1h, and -2b had single amplicon and were sequenced.

A

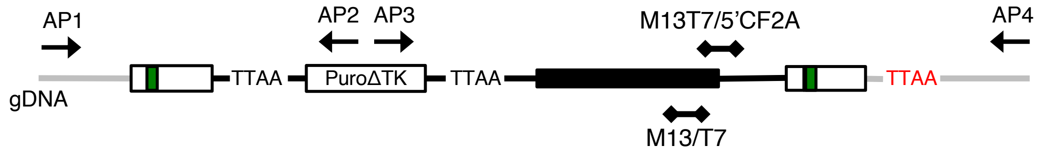

CRISPR Cas9n	left gRNA (5'>3')		right gRNA (5'>3')	gRNA offset
M13	TCGTTTTACAACGTCGTGAC tgg	T7	CGACTCACTATAGGGCGAAT tgg	22-bp
M13T7	GTCGTATTACGCGCGCTCAC tgg	5'CF2A	GCCTTGCAGGTCTCTGCTTC tgg	24-bp

B

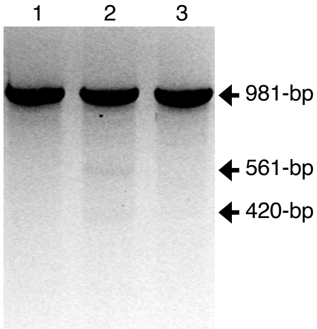

lane	Nuclease pair	Fragment size (bp)	
1	M13/T7	393	588
2	M13T7/5'CF2A	420	561
3	PBase	981	

Supplemental Fig S8 Assessment for plasmid DNA backbone-targeting CRISPR/Cas9n-gRNAs. (A) Targeting sites and sequence of CRISPR/Cas9n-gRNAs used for plasmid DNA backbone targeting. M13, T7, and M13T7 gRNAs are targeting plasmid DNA backbone, while 5' CF2A gRNA is targeting gDNA. (B) T7E1 assay for plasmid DNA backbone-targeting CRISPR/Cas9n-gRNAs. Each pair was transfected in Ic14 and the genomic DNA was harvested for PCR with f1 ori Rv/CF45 primer pair, followed by T7E1 assay. Each corresponding CRISPR/Cas9n-gRNAs pairs to the gel picture (left panel) is listed in the table with digested fragment sizes (lane 1 and 2, right tables). PBase was overexpressed as control (lane 3).

A I.c14 P8.13.11

Experiment	Cell numbers	Excision Treatment	FIAU (µM)	Picked	PB^- (-/test)	corr/seq
a	1.5 $x10^6$	Pair 2w (2.5 µg EA)	0.25	96	2/96	0/2
b	1.5 $x10^6$	M13/T7 (2.5 µg EA)	0.25	96	0/96	NA
c	1.5 $x10^6$	M13T7/5'CF2 (2.5 µg EA)	0.25	96	2/96	2/2
d	1.5 $x10^6$	Pbase (4.5 µg)	0.25	96	1/96	NA

B FIAU clone

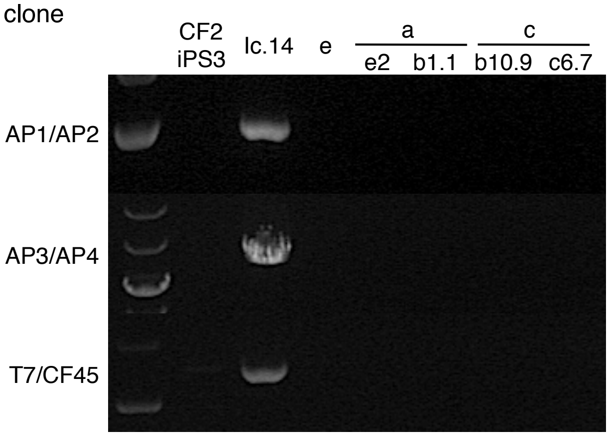

C

CF2-iPS3 I.c14$_{FIAU}$a-e2: W1282W (G>G) + 8-bp deletion/ W1282A (G>A)

I : ccagatcgatggtgtgtcttgggattcaataac........agtg**G**aggaaagcctttggagtgataccacag
N: ccagatcgatggtgtgtcttgggattcaataactttgcaacagtg**A**aggaaagcctttggagtgataccacag

CF2-iPS3 I.c14$_{FIAU}$a-b1: W1282A (G>A) + 6–bp insertion/ W1282? (G>?) + large insertion

I : ccagatcgatggtgtgtcttgggattcaataactttgcaa**INS**
N: ccagatcgatggtgtgtcttgggattcaataactttgcaacag**caacag**tg**A**aggaaagcctttggagtgata

CF2-iPS3 I.c14$_{FIAU}$c-b10: W1282W (G>G)/ W1282X (G>A)
CF2-iPS3 I.c14$_{FIAU}$c-c6: W1282W (G>G)/ W1282X (G>A)

I : ccagatcgatggtgtgtcttgggattcaataactttgcaacagtg**g**aggaaagcctttggagtgataccacag
N: ccagatcgatggtgtgtcttgggattcaataactttgcaacagtg**A**aggaaagcctttggagtgataccacag

Supplemental Fig S9 Excision of insertion and Negative selection with FIAU. (A) Summary of the excision efficacy for each strategy presented in Fig 5 followed by negative selection with FIAU instead of GCV. Ic14 (P8.13.11) was transfected with four treatments presented in Fig 5 and negatively selected with FIAU. (B) Confirmations of the removal of inserted plasmid DNA backbone using the insert specific primer pairs in all PB^- clones in treatment a, and c. (C) Sequence of clones screened from Ic14 clones with excised inserted backbone by treatment a (pair 2w), and c (M13T7/5'CF2A). Red capital letter shows *W1282X* mutation site. Both Ic14$_{GCV}$a clones have indel in corrected allele at upstream of *W1282X* mutation site shown as dot (deletion) or blue letter (insertion). Both two Ic14$_{GCV}$c clones have corrected and uncorrected allele without indels.

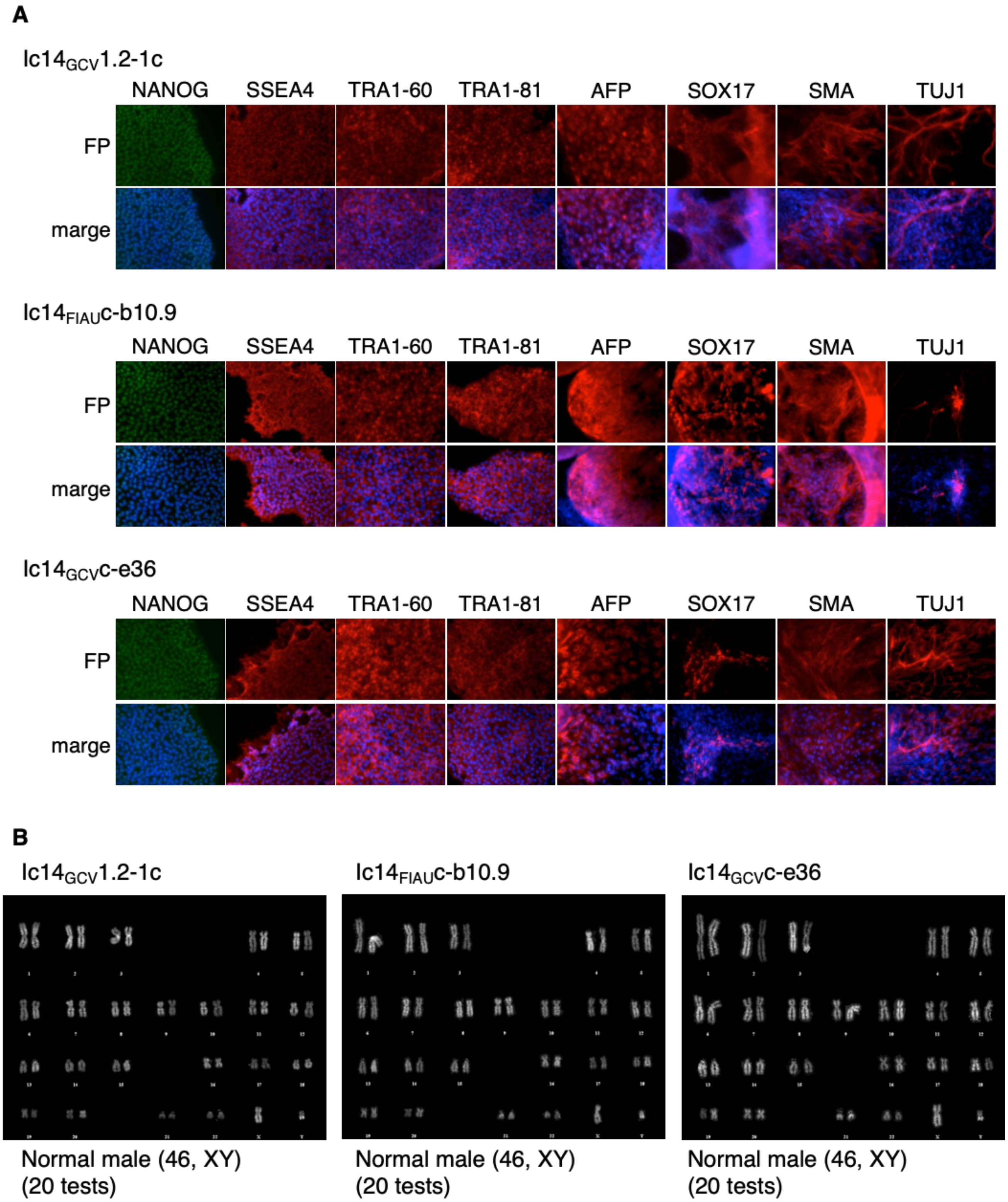

Supplemental Fig S10. Characterization of corrected CF2-iPS3 cells with removed insertion. (A, B) Characterization of representative corrected clones, Ic14GCV1.2-1c, Ic14FIAUc-b10.9, and Ic14GCVc-e36, by (A) immunocytochemical analysis for three germ layers and ES markers and (B) karyotyping.

Supplemental Table S1: Antibody list

PRIMARY	VENDOR (Catalog #)
Anti-Nanog antibody, mouse monoclonal IgG1	Abcam (ab62734) or BD Biosciences (560109)
Anti-SSEA3 antibody, rat monoclonal IgM	Millipore (MAB4303)
Anti-SSEA4 antibody, mouse monoclonal IgG3	Abcam (ab16287)
Anti-TRA1-60, mouse monoclonal IgM	Millipore (MAB4360) or BD Biosciences (560071)
Anti-TRA1-81, mouse monoclonal IgM	Millipore (MAB4381)
Anti-α-smooth muscle actin, mouse monoclonal IgG2a (mesoderm)	Sigma-Aldrich (A5228) or Thermo Scientific (MA5-11547)
Anti-Neuronal class III β-tubulin (TUJ1), mouse monoclonal IgG2a (ectoderm)	Covance (MMS-435P)
Anti-α-fetoprotein, mouse monoclonal IgG1 (endoderm)	R&D Systems (MAB1369) or Sigma-Aldrich (A8452)
Alexa Fluor 488 mouse anti-human Sox17	BD Biosciences (562205)
SECONDARY	VENDOR (Catalog #)
Alexa Fluor 488 goat anti-mouse IgG1	Invitrogen (A21121)
Goat anti-mouse IgG3-FITC	Sana Cruz Biotech (SC-3081)
Alexa Flour 546 goat anti-mouse IgM	Invitrogen (A21045)
Alexa Fluor 594 goat anti-rat IgM	Invitrogen (A21213)
Alexa Fluor 555 goat anti-mouse IgG	Invitrogen (A21424)
Alexa Fluor 594 goat anti-mouse IgG2a	Invitrogen (A21135)

Supplemental Table S2: Primer list for Analysis PCR

Analysis PCR		
	Name	5' > 3'
	CF2-AP1	AGTGCCACTAACTGTCAGCC
	AP2	GTCCTAAATGCACAGCGACG
	AP3	GCATTGTCTGAGTAGGTGTCATTC
	CF2-AP4	ACAAACTTGGGAGACTCACTTTG
	CF44	GGAATTGGACCCCTGATGCA
	CF45	TCCTTTTGCTCACCTGTGGTAT
	CF46	AGGGGTCCAATTCCTTATGGC
	CF47	CTAACTTGGAGGTCAGGCCAC
	CF48	CCAAGTTAGCAATCGCCAGGT
	P4	ACTGCCAAGTAGGAAAGTCCCATA
	P8	ATGGAGTTCCGCGTTACATAACTT
	P6r	ATCCGGCACCGGGCTTGCGGGTCATGCACCAG
	P7f	TCGAGCGGGTCACCGAGCTGCAAGAACTC
	T3	AATTAACCCTCACTAAAGGGAACAAAAGCTGG
	T7	GTAATACGACTCACTATAGGGCGAATTGGG
	f1ori Rv	CGTGAACCATCACCCTAATCAA

AP primers (analysis primers) can be used to screen for vector replacement and vector insertion events.
CF primers (CFTR primers) are complementary to CFTR sequences found in both vector and genomic DNA
P primers (puromycin primers) are used to for PCR amplification of the PuroΔTK cassette and immediately adjacent sequences.
T3 and T7 primers are complementary to the T3 and T7 promoter sequences in vector backbone

Supplemental Table S3: Primer list for Donor DNA construction

Donor DNA construction		
	Name	5' > 3'
Cloning	CF2A-2 fw	TTGCAGGTCTCTGCTTCTGG
	CF2A rv	GGAGGTGCTCCTGGCATTTTA
	CF2B fw	AGAACACAGAGTTGGGGCTC
	CF2B rv	AACTCTGGCCCACTTGGTTTT
Recombinant	P1-CF2A-F	TTGGCGCGCCTTGCAGGTCTCTGCTTCTGG
PCR	P1-CF2A-R	AATTTTACGCAGACTATCTTTCTAGGGTTAAAAAACAAAAGCCCAAGGCTC
	P2-CF2-F	CAGTGGGAGCCTTGGGCTTTTGTTTTTTAACCCTAGAAAGATAGTCTGC
	P2-R	GCGTACTTGGCATATGATACACTT
	P3-CF2A-F	ACAATATGATTATCTTTCTAGGGTTAACAGCTCTTTTTTGTTCCTGCT
	P3-CF2A-R	ATAAGAATGCGGCCGCGGAGGTGCTCCTGGCATTTTA
	P4-CF2A-R	AGCAGGAACAAAAAAGAGCTGTTAACCCTAGAAAGATAATCATATTGT
	P5-F	CACTAGTTAAAAGTTTTGTTACTTTATAGAAG
	P1-CF2B-F	TTGGCGCGCCAGAACACAGAGTTGGGGCTC
	P3-CF2B-R	ATAAGAATGCGGCCGCAACTCTGGCCCACTTGGTTTT

Supplemental Table S4: Oligo list for gRNA construction

gRNA Name		5' > 3'
CF2 Cas9n	Top	CACCGCCCAAGACACACACCATCGATC
	Bottom	AAACGATCGATGGTGTGTCTTGGGC
CF2 Cas9n1	Top	CACCGAGCCTTTGGAGTGATACCAC
	Bottom	AAACGTGGTATCACTCCAAAGGCTC
CF2 Cas9n2	Top	CACCGCAATAACTTTGCAACAGTGA
	Bottom	AAACTCACTGTTGCAAAGTTATTGC
CF2 Cas9nw2	Top	CACCGCAATAACTTTGCAACAGTGG
	Bottom	AAACCCACTGTTGCAAAGTTATTGC
CF2 Cas9n3	Top	CACCGCAACAGTGAAGGAAAGCCTT
	Bottom	AAACAAGGCTTTCCTTCACTGTTGC
CF2int gR-A	Top	CACCGCAAGGCTCCCACTGTAAATT
	Bottom	AAACAATTTACAGTGGGAGCCTTGC
CF2int gR-B	Top	CACCGCTTTTTTGTTCCTGCTTCAG
	Bottom	AAACCTGAAGCAGGAACAAAAAAGC
CF2int gR-C	Top	CACCGTGTTAAAAAACAAAAGCCCA
	Bottom	AAACTGGGCTTTTGTTTTTTAACAC
CF2int gR-9	Top	CACCGGGAGGTCAGGCCACTGAAGC
	Bottom	AAACGCTTCAGTGGCCTGACCTCCC
CF2int gR-2	Top	CACCGCCTCCAAGTTAGCAATCGCC
	Bottom	AAACGGCGATTGCTAACTTGGAGGC
M13 C9n	Top	CACCGTCGTTTTACAACGTCGTGAC
	Bottom	AAACGTCACGACGTTGTAAAACGAC
T7 C9n	Top	CACCGCGACTCACTATAGGGCGAAT
	Bottom	AAACATTCGCCCTATAGTGAGTCGC
M13T7 C9n	Top	CACCGGTCGTATTACGCGCGCTCAC
	Bottom	AAACGTGAGCGCGCGTAATACGACC
5'CF2A C9n	Top	CACCGGCCTTGCAGGTCTCTGCTTC
	Bottom	AAACGAAGCAGAGACCTGCAAGGCC

SUPPLEMENTAL MATERIALS AND METHODS

Production of Recombinant Retrovirus and Cell Reprogramming

CF-iPSCs were generated by retroviral transduction with the canonical transcription factors OCT4, KLF4, SOX2, and c-MYC as described previously (Suzuki et al. 2016b) and according to guidelines developed by the Stem Cell Research Oversight (SCRO) Committee at the CPMC Research Institute (CPMCRI) and the UCSF Gamete and Embryonic Stem Cell Research (GESCR) Committee. Individual recombinant retroviruses were generated by co-transfecting individual retroviral plasmids (pMXs) that contain human reprogramming factors (Takahashi et al. 2007), GFP (Addgene Inc., Cambridge, MA), vesicular stomatitis virus G protein (VSV-G) and the gag-pol into HEK293T cells. CFNPF7 fibroblasts passage 8 (P8) were transduced with retrovirus containing human reprograming factors and reprogrammed. Retroviral transduced CFNPF7 primary cells were grown on MEF feeder cells in hESC medium as described previously (Suzuki et al. 2016b) until candidate CF-iPSCs clones were identified and isolated. Candidate clones were then expanded either on MEF feeder cells in hESC medium or on Matrigel (BD Biosciences, San Jose, CA) in mTeSR1 medium (StemCells Inc., Vancouver, BC, Canada). Individual CF-iPSCs colonies were grown and initially subcultured by mechanical isolation, with subsequent subculture using enzymatic dissociation with Dispase (StemCell Inc.), or non-enzymatic dissociation with ReLeSR (StemCell Inc.).

Assessment of modification efficiency by CRISPR/Cas9n-gRNAs using the T7E1 Assay:

The T7E1 assay is used to detect heteroduplex DNA molecules and indirectly detect NHEJ formation in cells from DSB-inducing events like Crispr/Cas9 DNA cleavage. The T7E1 endonuclease has strand cutting activity that recognizes and cleaves at the base of heteroduplex DNA structures formed by the annealing of indel containing DNA strands with unmodified DNA strands. The resulting T7E1 cleaved products are resolved by agarose gel electrophoresis and quantified (Vouillot et al 2015).

CFBE41o- cells were nucleofected with CRISPR/Cas9n-gRNAs and genomic DNA was harvested to test the CRISPR/Cas9n-gRNAs modification efficiency using T7 endonuclease I assay (T7E1 assay). Briefly, CFBE41o- cells were nucleofected with 4D Nucleofector X, SF cell line solution and Program DS120 (Lonza, Walkersville, MD) by following the manufacturer's protocol. 2×10^5 CFBE41o- cells were nucleofected with 0.4 µg of each pairs of CRISPR/Cas9n-gRNAs, and genomic DNA was harvested using GeneJet kit (Thermo Fisher scientific) at Day 5 to 7 post-transfection. Regions targeted by CRISPR/Cas9n-gRNAs were amplified with primer pair CF46/CF47 for exon 23 targeting and f1ori rev/CF45 for plasmid DNA backbone targeting, and PCR product was purified by silica-based nucleotide purification technique, NucleoSpin Gel and PCR Clean-up (Macherey-Nagel, Bethlehem, PA). Purified product was denatured on a thermocycler at 95 °C for 10 min and re-annealed slowly by ramping temperature down from 95 °C to 25 °C. Annealed product was digested by T7E1 at 37 °C for 30 min to 1 hr and ran on 2% gel. When imaging the gel, all bands were imaged without saturation to quantify the band intensities using Image J (U. S. National Institutes of Health, Bethesda, Maryland, USA, http://imagej.nih.gov/ij/). The percentage of NHEJ was calculated from the band intensities according to the following formula (Guschin et al., 2010) as the modification efficiency by CRISPR/Cas9n-gRNAs:

$$f_{cut} = \frac{CleavageBand_1 + CleavageBand_2}{CleavageBand_1 + CleavageBand_2 + UncleavedBand}$$

$$\%NHEJ = 100 * \left(1 - \sqrt{1 - f_{cut}}\right)$$

Identification of donor plasmid DNA insertion: To identify the high molecular weight (HMW) band that was observed during HR event screening by AP3/4 PCR representing 3' end of targeting site (Fig 1B), HMW AP3/4 amplicons were further investigated by PCR and restriction enzyme digestion. Briefly, HMW PCR products were separated by 0.8% of low melting temperature agarose gel (Thermo Fisher Scientific) and gel-purified with NucleoSpin Gel and PCR clean kit (Macherey Nagel). The gel-purified HMW PCR products were used as the template DNA to run PCR with primers AP3/CF44, AP3/T3, CF46/CF47, and T7/CF45 to identify HMW bands. For restriction enzyme digestion, AP3/CF44 PCR product was gel-purified as described above, and digested with NotI and/or ScaI at 37 °C overnight. Single- or double-digested samples were separated on a 2% agarose gel containing ethidium bromide and visualized under UV light.

Reference

Guschin, D.Y., Waite, A.J., Katibah, G.E., Miller, J.C., Holmes, M.C., and Rebar, E.J. (2010). A rapid and general assay for monitoring endogenous gene modification. *Methods Mol Biol* 649**,** 247-256.

Suzuki, S., Sargent, R.G., Illek, B., Fischer, H., Esmaeili-Shandiz, A., Yezzi, M.J., Lee, A., Yang, Y., Kim, S., Renz, P., Qi, Z., Yu, J., Muench, M.O., Beyer, A.I., Guimaraes, A.O., Ye, L., Chang, J., Fine, E.J., Cradick, T.J., Bao, G., Rahdar, M., Porteus, M.H., Shuto, T., Kai, H., Kan, Y.W., and Gruenert, D.C. (2016b). TALENs Facilitate Single-step Seamless SDF Correction of F508del CFTR in Airway Epithelial Submucosal Gland Cell-derived CF-iPSCs. *Mol Ther Nucleic Acids* 5**,** e273.

Takahashi,K., Tanabe, K., Ohnuki, M., Narita, M., Ichisaka, T., Tomoda, K., and Yamanaka**,** S. (2007). Induction of Pluripotent Stem Cells from Adult Human Fibroblasts by Defined Factors. *Cell* 131, 861-872

Vouillot, L, Thelie, A, and Pollet, N. (2015) Comparison of T7E1 and surveyor mismatch cleavage assays to detect mutations triggered by engineered nucleases. *G3: Genes, Genomes, Genetics* 5, 407-415
